# Comparative Metagenomic Analysis of Electrogenic Microbial Communities in Differentially Inoculated Swine Wastewater-Fed Microbial Fuel Cells

**DOI:** 10.1155/2017/7616359

**Published:** 2017-10-12

**Authors:** Irina V. Khilyas, Anatoly A. Sorokin, Larisa Kiseleva, David J. W. Simpson, V. Fedorovich, Margarita R. Sharipova, Mami Kainuma, Michael F. Cohen, Igor Goryanin

**Affiliations:** ^1^Institute of Fundamental Medicine and Biology, Kazan (Volga Region) Federal University, Kazan, Russia; ^2^Institute of Cell Biophysics, Russian Academy of Sciences, Pushchino, Moscow Region, Russia; ^3^Moscow Institute of Physics and Technology, Dolgoprudny, Moscow Region, Russia; ^4^Biological Systems Unit, Okinawa Institute of Science and Technology, Okinawa, Japan; ^5^Department of Biology, Sonoma State University, Rohnert Park, CA, USA; ^6^School of Informatics, University of Edinburgh, Edinburgh, UK; ^7^Tianjin Institute of Industrial Biotechnology, Tianjin, China

## Abstract

Bioelectrochemical systems such as microbial fuel cells (MFCs) are promising new technologies for efficient removal of organic compounds from industrial wastewaters, including that generated from swine farming. We inoculated two pairs of laboratory-scale MFCs with sludge granules from a beer wastewater-treating anaerobic digester (IGBS) or from sludge taken from the bottom of a tank receiving swine wastewater (SS). The SS-inoculated MFC outperformed the IGBS-inoculated MFC with regard to COD and VFA removal and electricity production. Using a metagenomic approach, we describe the microbial diversity of the MFC planktonic and anodic communities derived from the different inocula. Proteobacteria (mostly Deltaproteobacteria) became the predominant phylum in both MFC anodic communities with amplification of the electrogenic genus* Geobacter* being the most pronounced. Eight dominant and three minor species of* Geobacter* were found in both MFC anodic communities. The anodic communities of the SS-inoculated MFCs had a higher proportion of* Clostridium* and* Bacteroides* relative to those of the IGBS-inoculated MFCs, which were enriched with* Pelobacter*. The archaeal populations of the SS- and IGBS-inoculated MFCs were dominated by* Methanosarcina barkeri* and* Methanothermobacter thermautotrophicus*, respectively. Our results show a long-term influence of inoculum type on the performance and microbial community composition of swine wastewater-treating MFCs.

## 1. Introduction

Livestock farming constitutes an important agricultural sector of many countries but produces considerable amounts of organic wastes that require proper treatment and disposal. The rapidly growing pig farming industry generates high-strength wastewater containing organic compounds, ammonia, phosphates, odorous gases, suspended solids, and pathogens [1]. Treating swine wastewater is especially difficult where land is limited and pig farming facilities occur in close proximity to population centers, such as in Okinawa, Japan. The lack of available land for application of swine wastewater (SW) as a fertilizer and potential for contamination of surface and ground water sources underscore the need to employ thorough treatment of SW.

Common methods of treating SW include aerobic oxidation ponds, lagoons, anaerobic digestion, and constructed wetlands [2]. Bioelectrochemical systems such as microbial fuel cells (MFCs) are promising new technologies for efficient removal of organic compounds in wastewaters. Inside the confined anaerobic chamber of an MFC, a consortium of bacteria catalyze oxidation reactions, depositing electrons on the anode by a variety of means, such as directly via outer membrane proteins or conductive pili or indirectly via secretion and recycling of redox-active molecules [3].

A primary target of SW treatment is a set of volatile fatty acids (VFAs) largely responsible for its noxious odor [4]. The presence of VFAs in an MFC substrate can increase the electrogenic performance of its anodic microbial biofilm [5]. Laboratory-scale single batch-loaded MFCs have been shown to dramatically lower malodorous compounds (primarily VFAs) as well as other constituents present in SW [4].

One important determinant of MFC reactor performance is the composition of the microbial community in the anodic chamber [6]. For obtaining maximal initial power production, the anodic biofilm of an existing MFC has been shown to serve as a better inoculum than anaerobic sludge, but we know of no study that assesses inoculum performance relative to pollutant removal criteria [7]. To this end, we sought to determine whether a microbial community already familiar with a SW substrate would perform better in an MFC than a distinct beer waste-digesting anaerobic sludge, assessing treatment performance and microbial community composition.

Previous studies have assessed microbial community composition in SW-fed MFCs utilizing denaturing gel gradient electrophoresis, while a more recent study has utilized high-throughput amplicon sequencing to examine influences of external resistance and hydrodynamics on the MFC microbiome [8, 9]. Using a metagenomic approach here we describe the microbial diversity of the MFC planktonic and anodic communities derived from the different inocula. Clustering of microbial communities based on dominant bacterial genera indicates that the nature of the inoculum is an important influence on the ultimate composition of microbial communities and performance of MFCs.

## 2. Materials and Methods

### 2.1. MFC Configuration and Operation

The internal MFC chamber contained two anodes (approximately 6 × 8 cm), suspended 2-3 mm off the bottom of the chamber, composed of a layer of conductive carbon cloth to which 2 mm average size activated carbon granules were bound with conductive glue to provide more surface area. The granules had been prepared from birch precursor and were pretreated with a neutral red catalyst. The two cathodes were graphite plates (3 mm thick; 60% porosity) sprayed on the liquid-facing side with an aqueous 5% Fumion membrane polymer (Fumatech, Bietigheim-Bissingen, Germany), while activated carbon granules [treated with iron(II) phthalocyanine] were mechanically pressed to the air-facing side using netting frame. The cathode extended into a bath containing an electrolyte solution (maintained at pH 3 with regular additions of 0.1 N HCl).

The anode and cathode electrodes were connected with a multichannel logger (Graphtec Midi LOGGER GL820, Japan) for daily voltage measurements. The corresponding electric current was calculated using Ohm's law (*V* = IR). Power density was obtained according to *P* = *IV*/*A*, where *I* is the current, *V* is the voltage, and *A* is the projected surface area of the cathode. Polarization and power curves, obtained by changing external resistances (from 0 Ω to 2100 Ω) in open circuit when the values had stabilized at each resistance, indicated an internal resistance of 70 Ω within the MFCs (Figure S1 in Supplementary Material available online at https://doi.org/10.1155/2017/7616359).

For inoculation of the MFCs, swine wastewater sludge (SS) containing suspended scrapings from the bottom of the SW holding tank was collected from a local pig farm (Okinawa Livestock and Grassland Centre, Nago, Japan) and industrial granular brewery sludge (IGBS) from a wastewater-treating UASB reactor (Orion Brewery, Nago, Japan). The inocula were not chemically modified or diluted though the SS inoculum was filtered through a 1 mm stainless steel mesh.

To allow for microbial biofilm formation, two MFCs were inoculated with SS and two with IGBS, heretofore referred to as SS-MFC and IGBS-MFC, and allowed to sit for 3 days in open-circuit mode at room temperature (24°C). The MFCs were then provided undiluted SW in fed-batch mode to achieve a 24 h HRT. Regular feeding and monitoring of electrical performance began 13 d following inoculation of the MFCs, coincident with the switch to closed-circuit mode.

SW for use as MFC feed was stored at 4°C. To remove large particles, the raw SW was sieved through a 0.50 mm mesh (Nylon monofilament). SW feed was diluted with distilled water to adjust the chemical oxygen demand (COD) to 3.5–7.4 g O_2_ L^−1^ and the hydraulic retention time (HRT) set to 1 or 2 d over the course of the experiment. Wastewater was added to the MFCs semicontinuously using a peristaltic pump (Masterflex L/S Precision Pump, Cole-Parmer, USA) set to a 6 ml min^−1^ flow rate. Operational parameters for the MFCs over the course of the 67-day experiment are summarized in Table S1.

### 2.2. Chemical Analyses

Sampling of MFC inflow and outflow was performed every 24 h. COD, volatile fatty acids (VFA), ammonia nitrogen (NH_3_-N), and total phosphorus (PO_4_^3−^-P) determinations were measured using the HACH TNTplus Chemistries (HACH Company, Loveland, CO). Total COD of inlet swine wastewater and MFC-treated effluent was measured without filtration. pH was measured with a pH meter (Horiba D-51, Japan).

### 2.3. Chromatography

Specific VFA compounds were quantified using an Agilent 7890A gas chromatograph connected to a LECO Pegasus 4D TOF mass spectrometer. Separation of VFA was performed using a Stabilwax-DA (30 m, 0.25 mm ID, and 0.25 *μ*m) column, using helium as carrier gas at 1.11 ml min^−1^ flow for the entire run. Method development was performed using Supelco WSFA-2 Mix to obtain retention index (RI) calibration and quantification calibration curve. Approximately 1 ml of sample was transferred through a 0.22 *μ*m filter to a glass autosampler vial. A 1 : 20 split liquid injection (1 *μ*l volume) was injected, with the injection port set at 250°C, 1 ml min^−1^ septum purge flow. The gradient temperature protocol was 2 min at 100°C followed by an increase to 145°C at a rate of 20°C min^−1^, holding at 145°C for 6 min, followed by an increase to 205°C at 20°C min^−1^ and holding this temperature for 4 min. The mass spectrometer was set with 35 to 145 Da mass scan range, 5 spectra sec^−1^ acquisition rate, and −70 V electron energy. Ion source and transfer line temperature was 250°C. Data processing (deconvolution, identification, and quantification) was done using LECO ChromaTOF version 4.50.8 software. Acetic acid (99.99% purity), butyric acid (99.5%), 2-ethylbutyric acid (99%), hexanoic acid (99.5%), isovaleric acid (99%), isobutyric acid (99.5%), octanoic acid (99.5%), propionic acid (99.8%), sulfuric acid (99.9%), and valeric acid (99.8%) standards were purchased from Sigma-Aldrich, Japan.

### 2.4. Microbial Diversity Analysis

DNA was isolated from swine wastewater, inoculum sludges, anodic biofilms (carbon felt and carbon granules), and planktonic samples of each MFC using PowerMax soil DNA isolation kit (MO BIO laboratories, Inc.). DNA quality was evaluated by the Agilent 2100 Bioanalyzer system. A DNA library was constructed for shotgun sequencing and a 150 paired-end sequencing reaction was performed on MiSeq platform (Illumina, San-Diego, CA, USA).

The sequencing data were uploaded to the MG-RAST server as FASTAQ files for processing, primary analysis, and storage.* Sus scrofa* (pig) genome sequences were marked for exclusion during data submission. Primary submission data and results of the MG-RAST pipeline are available publicly (project mgp19536). The MG-RAST representative hit organism abundances calculation was performed against the SEED database at the level of genera, based on a maximum *e*-value of 1 × 10^−5^, minimum identity cut-off of 60%, and minimum sequence alignment of 15. Abundance data were downloaded as TSV files for further analysis. The representative hit data were downloaded from MG-RAST server via MGRASTer package [https://github.com/braithwaite/MGRASTer/] in R 3.1 environment. Abundance analysis was performed in metagenome Seq package [10] and ordination analysis was performed with phyloseq R packages [11]. Krona taxonomic community profiles were built by MG-RAST and stored as an image.

## 3. Results

We applied an integrated approach to investigating the effect of two distinct inoculums on performance of MFCs treating SW, comparing source- and site-dependent differences in the diversity of the microbial community, electricity production, and removal of organics.

### 3.1. MFC Performance Characteristics

The SS-MFC pairs outperformed the IGBS-MFCs pairs in regard to electricity generation (Figure S2) and removal of COD and VFA from the SW feed (Table 1; Figure S3) while operating on a 48 h HRT. Both MFC pairs displayed negligible removal phosphate (Table 1, Figure S4), whereas the SS-MFCs performed better than the IGBS-MFCs at removing ammonia (Table 1, Figure S5). Over the 67 d course of the experiment, the SS-MFC COD removal rate of 2.65 ± 0.11 mg O_2_ L^−1^ d^−1^ was slightly but significantly higher than the IGBS-MFC rate of 2.26 ± 0.17 mg O_2_ L^−1^ d^−1^ (*p* = 0.02), while their respective VFA removal rates of 0.76 ± 0.06 mg L^−1^ d^−1^ and 0.66 ± 0.05 mg L^−1^ d^−1^ did not differ significantly (*p* = 0.27; means ± SE, *n* = 4). Electrical output of the MFC pairs remained relatively stable over the course of the experiment with the current density of the SS-MFCs (56.6 ± 2.4 mA m^−2^) being consistently higher than that of the IGBS-MFCs (43.5 ± 6.2 mA m^−2^) (means ± SD, *n* = 43; Figure S2).

Changes in the concentrations of straight-chain (acetic, propionic, butyric, valeric, and hexanoic) and branched chain (isobutyric, isovaleric) VFAs were monitored (Table 2). Of these, propionic acid was found at the highest concentration in the SW. Passage through the SS-MFC removed >90% of all monitored VFAs, except for propionic acid, which was dissipated by 85.9%, and outperformed the removal rate of IGBS-MFC for all VFA tested. Predominance of propionic or acetic acids among VFAs in MFC-treated SW effluent has been previously shown [4, 12]. Several aromatic ring compounds (phenols and indoles) can contribute to the odor of SW [4]; however, we detected only two aromatic compounds (p-cresol and phenol) at negligible concentrations and indoles were not found (results not shown).

### 3.2. Metagenomic Analysis of Microbial Communities

#### 3.2.1. Inocula

**Figure 1:**
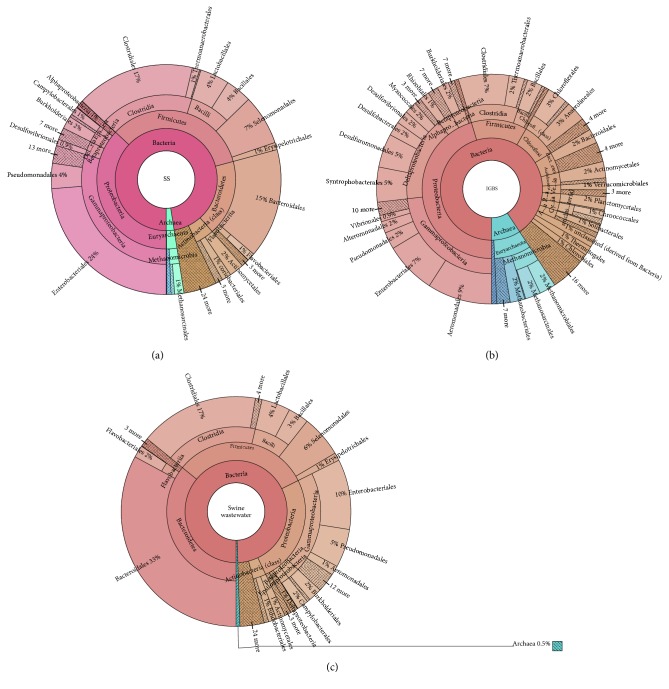
Summary of the microbial community profiles in the multilevel Krona diagrams. Krona plots visualizing taxonomic hierarchies of the microbial communities of (a) swine sludge (SS), (b) industrial granular brewery sludge (IGBS), and (c) swine wastewater.

Over 98% and 91% of genes were affiliated with the domain Bacteria, and only 2% and 9% of genes were represented by Archaea for the SS and IGBS inoculums, respectively (Figures 1(a) and 1(b)). 30% Gammaproteobacteria (dominant genus Enterobacteriaceae (24%)) were the most abundant in SS inoculum, whereas 23% Gammaproteobacteria, dominated by the genera Aeromonadaceae (9%) and Enterobacteriaceae (7%), were identified in IGBS inoculum (Figures 1(a) and 1(b)). Deltaproteobacteria were represented by the dominant families Desulfovibrionaceae (0.8%) in SS inoculum and Geobacteraceae (3%) and Syntrophaceae (3%) in IGBS inoculum (Figures 1(a) and 1(b)).

The phylum Firmicutes (34% (SS) and 13% (IGBS)) was represented by Clostridia and Bacilli classes in both inoculums (Figures 1(a) and 1(b)). The most abundant members of phylum Bacteroidetes (18%) were identified as Prevotellaceae (8%) and Bacteroidaceae (5%) in SS inoculum (Figure 1(a)). Despite the low content of Bacteroidetes in IGBS inoculum (5%), the diversity of bacterial families was similar to that of the SS inoculum (Figure 1(b)). On the other hand, IGBS inoculum was enriched by Chloroflexi (8%), with dominant members Anaerolineaceae (3%) and Chloroflexaceae (3%) (Figures 1(a) and 1(b)). Actinobacteria (Actinomycetales) was found to be relatively abundant in both inoculums (Figures 1(a) and 1(b)). Cyanobacteria (Chroococcales) with abundance <0.5% were detected in SS inoculum, while 2% were identified in IGBS inoculum (Figures 1(a) and 1(b)).

Phylum Archaea was more abundant in the IGBS inoculum (9%) compared to the SS inoculum (2%) (Figures 1(a) and 1(b)). Analysis of two inoculums showed that Euryarchaeota (Methanosarcinales (1%) and Crenarchaeota (Desulfurococcales)) were represented in both inoculums (Figures 1(a) and 1(b)). Thus, two types of inoculums were analyzed in detail to investigate formation of electrogenic microbial communities within MFCs having effective treatment and degradative ability.

#### 3.2.2. Swine Wastewater

The microbial community analysis of SW showed that Bacteroidetes (36%), Firmicutes (32%), Proteobacteria (25%), and Actinobacteria (3%) and 24 classes of Bacteria with relative abundance >1% were present (Figure 1(c)). The Proteobacteria were composed of Gammaproteobacteria (19%) (predominantly Enterobacteriaceae (10%)), Epsilonproteobacteria (2%) (with Campylobacteraceae (1%)), Deltaproteobacteria (1%) (with Desulfovibrionaceae (0.4%)), and Alphaproteobacteria (0.9%) (with Rhizobiales (0.4%)) (Figure 1(c)). Phyla Firmicutes and Bacteroidetes of the SW microbial community had a distribution of dominant members similar to the SS inoculum (Figures 1(a)–1(c)). Archaeal communities representing 0.5% of the total detected bacteria were dominated by classes of methanogens Methanomicrobia and Methanobacteria (Figure 1(c)).

#### 3.2.3. MFC Anodic Microbial Communities

**Figure 2:**
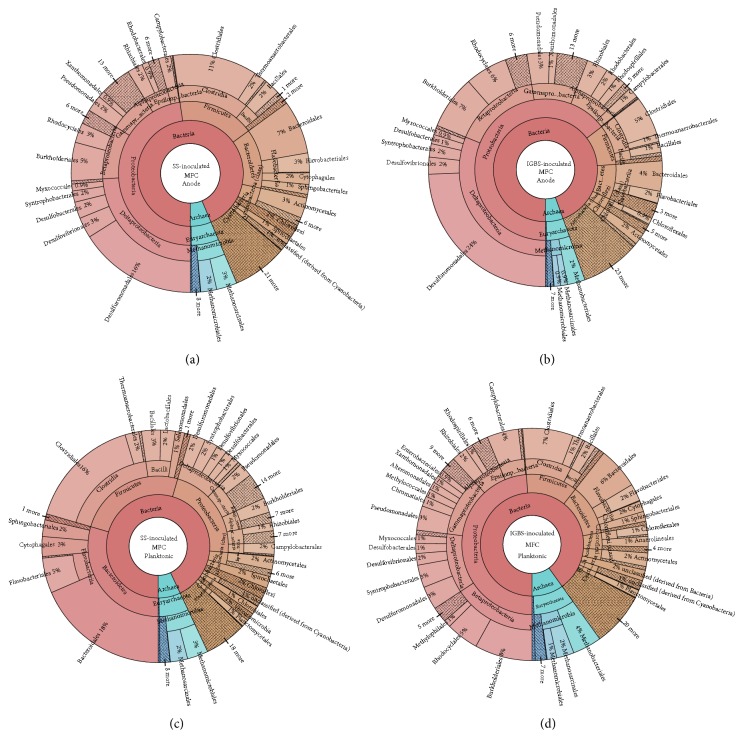
Summary of the anodic and planktonic microbial community profiles in multilevel Krona diagrams. Krona plots visualizing taxonomic hierarchies of the microbial communities of (a) swine sludge-inoculated MFC anode, (b) industrial granular brewery sludge-inoculated MFC anode, and (c) swine sludge-inoculated MFC planktonic contents, and (d) industrial granular brewery sludge-inoculated MFC planktonic contents.

The microbial diversity of anodic communities was similar between the SS-MFCs and IGBS-MFCs at the level of genera (Figure 2). Deltaproteobacteria reached up to 24% and 31% of the total microbial population on the anodes of SS-MFCs and IGBS-MFCs, respectively (Figures 2(c) and 2(d)). Among the Deltaproteobacteria class,* Geobacter* (14% and 20%) was identified as the most highly abundant genus on both MFC anodes.

The Gammaproteobacteria, common in both inocula (7% of SS and 8% of IGBS) and SW (30%), were substantially less represented on the anodes of both MFCs (Figures 2(c) and 2(d)). Particular declines in the Enterobacteriaceae led to higher relative levels of Moraxellaceae* (Acinetobacter)*, Pseudomonadaceae* (Pseudomonas),* and Xanthomonadaceae* (Xanthomonas)* among the Gammaproteobacteria in the anodic community of SS-MFCs and Pseudomonadaceae* (Pseudomonas)* and Xanthomonadaceae* (Xanthomonas)* in the anodic community of IGBS-MFCs (Figures 2(c) and 2(d)). Known electrogenic bacteria* Shewanella (*Shewanellaceae) were found on the anodes of both MFCs (Figures 2(c) and 2(d)).

Phylum Firmicutes (Clostridia and Bacilli) occupied only 13% and 9% of the total microbial population in SS-MFC and IGBS-MFC anodic communities, less compared to the inocula and SW (Figures 2(c) and 2(d)). Slight increases in the proportion of Bacteroidetes members (Bacteroidales and Flavobacteriales) (19%) were observed in the SS-MFC anodic communities compare to the inoculum (Figures 2(c) and 2(d)). Members of phylum Chloroflexi (*Roseiflexus* and* Anaerolinea*) were enriched in the population of anodic microbial community of SS-MFC, whereas that of the IGBS-MFC had less Chloroflexi (Figure 2). Slight enrichment of facultative heterotrophic Cyanobacteria genera (*Cyanothece, Synechococcus,* and* Nostoc*) on anodes of both MFCs was detected (Figures 2(c) and 2(d)).

Populations of Archaea significantly increased only on the anode of SS-MFCs (6%) compared to SS inoculum (2%) and SW (0.5%) (Figures 2(c) and 2(d)). The most abundant genera of Archaea were* Methanosarcina* on anode of SS-MFCs and* Methanothermobacter* on anode of IGBS-MFCs (Figures 2(c) and 2(d)).

#### 3.2.4. MFC Planktonic Microbial Communities

Analysis of the SS-MFC planktonic community showed that phyla Bacteroidetes (30%), Firmicutes (25%), Proteobacteria (22%), Actinobacteria (3%), and Archaea (7%) were highly abundant (Figure 2(a)). The dominant Gammaproteobacteria in inoculum and SW shifted to the Deltaproteobacteria in the MFC planktonic microbial communities (Figures 1(a)–1(c) and 2(a)). Members of phylum Archaea were enriched in the planktonic population similar to population of MFC anodic surface (Figures 2(a) and 2(b)). The planktonic community of the IGBS-MFCs was similar to their anodic microbial community (Figures 2(b)–2(d)). Dominant phyla Proteobacteria (49%), Firmicutes (12%), Bacteroidetes (12%), Chloroflexi (4%), Archaea (10%), and Actinobacteria (2%) were found in the planktonic community of IGBS- MFCs (Figure 2).

#### 3.2.5. Similarity- and Phylogeny-Based MFC Microbial Community Profiling

**Figure 3:**
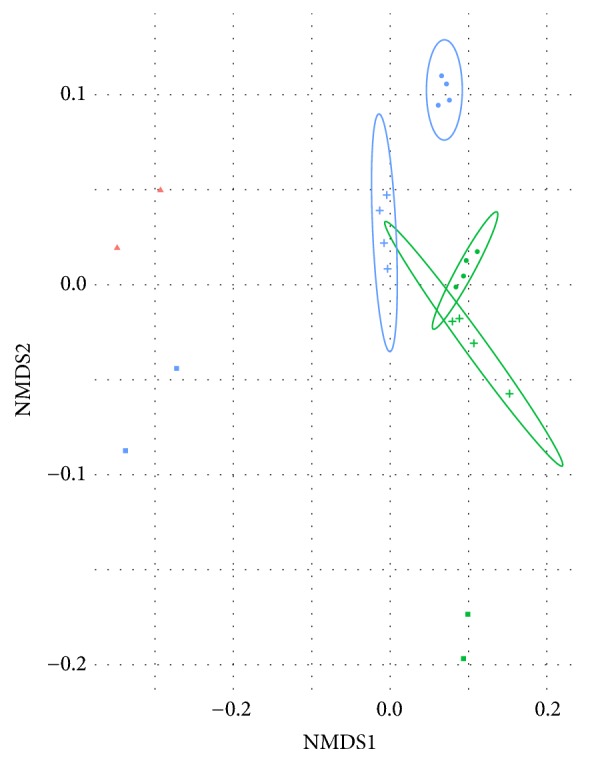
Ordination plots of a nonmetric multidimensional scaling (NMDS) for the microbial communities from SW (inflow) and SS- and IGBS-inoculated MFCs. Blue color indicates microbial communities derived from SS, green color indicates microbial communities derived from IGBS, and red color indicates microbial communities derived from SW (circles, anodic microbial communities; crosses, planktonic microbial communities; squares, microbial communities of inoculums; triangles, SW microbial community). NMDS was based on Bray-Curtis distances of prokaryotic species abundance.

To determine the relationship between MFC anodic and planktonic microbial communities, swine wastewater, and inocula, a two-dimensional ordination plot based on taxonomy was created (Figure 3). Statistically significant dissimilarities were observed across the SW and anodic and planktonic communities of both MFCs.

Each sample type can be seen to form a distinct cluster with the IGBS-MFC anodic and planktonic communities overlapping and the SS-MFC anodic and planktonic communities in close proximity. The SS and IGBS inoculum communities are well separated from each other. Thus, anodic and planktonic communities of IGBS-MFCs and SS-MFCs clustered close to one another, while SW samples did not (Figure 3).

A heat map of dominant bacterial genera based on a hierarchical clustering analysis was created to confirm the similarity and differences between the MFC anodic and planktonic microbial communities, swine wastewater, and inocula (Figure 3). The planktonic MFC communities have a high similarity to their inoculum communities. These planktonic-inoculum clusters form a secondary cluster with each other. The MFC anodic communities form their own distinct cluster which contains* Geobacter* spp., a well-known genus of electrogenic bacteria. The SW community differed from all microbial communities and formed a separate cluster (Figure 3). Clustering of microbial communities based on dominant bacterial genera indicates that the electrogenic communities in the MFC developed from their inocula.

#### 3.2.6. Diversity of Dominant Microbial Species in MFC Anodic Microbial Communities

**Figure 4:**
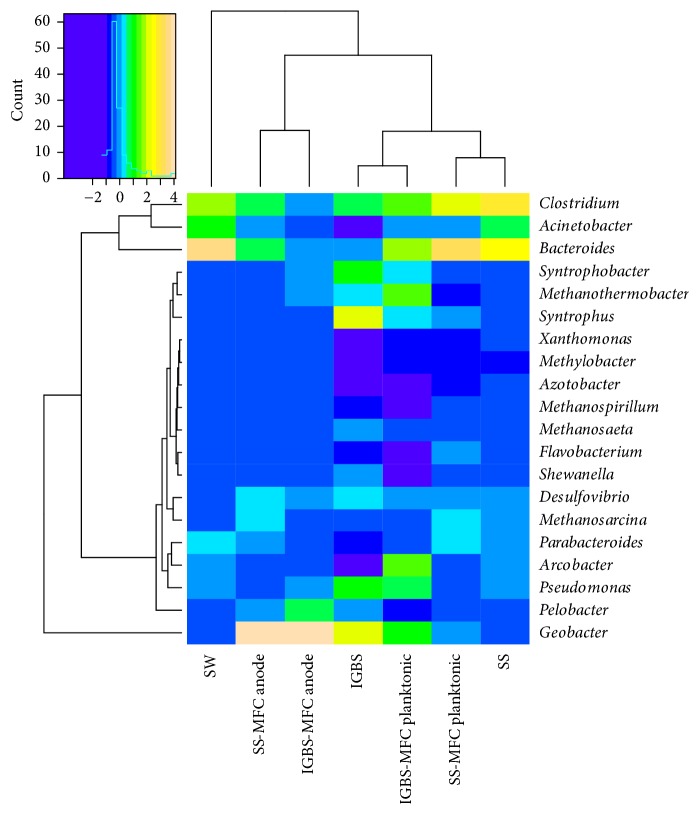
Heat map diagram visualizes the dominant bacterial and archaeal genera in the microbial community profiles. Bottom represents the different samples.

Detailed analyses revealed five abundant genera of Proteobacteria enriched on the anodes of MFCs. The genus* Geobacter* was represented by eight predominant and three minor species in both MFC anodic communities (Figure 4). Highly abundant* Pelobacter* genus* (Pelobacter propionicus, Pelobacter carbinolicus)* was identified in both anodic communities of MFCs (Figure 4). The diversity observed within the genus* Desulfovibrio* was significant (Figure 4). Gammaproteobacteria were represented by six dominant bacterial genera in the anodic communities of the SS-MFCs and IGBS-MFCs. The* Acinetobacter* genus was represented by four abundant species in anodic microbial communities of both MFCs. Diversity of* Pseudomonas* members associated with MFCs anodes was tremendous. Among them* Pseudomonas fluorescens* and* Pseudomonas aeruginosa* were the most abundant species in the anodic biofilms of the IGBS-MFCs and SS-MFCs, respectively. The* Azotobacter* genus was dominated by* Azotobacter vinelandii* on anodes of both MFCs. Six abundant species of* Xanthomonas* genus (dominant* Xanthomonas campestris*) were identified in the anodic biofilms of both MFCs. Twenty different members of* Shewanella* genus were found in anodic biofilms of both MFCs and* Shewanella baltica* was the most abundant specie among them. One member of* Methylobacter* genus* (Methylobacter tundripaludum)* was enriched on anodes of both MFCs (Figure 4).

Among Firmicutes, over 50 species of the genus* Clostridium* were identified from the MFC anodes, including 31 abundant species (dominant* Clostridium thermocellum*) and 9 relatively abundant species.* Flavobacterium johnsoniae* and* Flavobacterium psychrophilum, Bacteroides fragilis,* and* Parabacteroides distasonis* were the three dominant bacterial species among Bacteroidetes in the anodic communities of both MFCs. Acetoclastic methanogens (*Methanosarcina barkeri* and* Methanothermobacter thermautotrophicus*) belonging to the domain of Archaea were identified in MFC anodic and planktonic populations (Figure 4).

## 4. Discussion

This study demonstrated the compositions and phylogenetic distributions of SW, inocula, anodic, and planktonic microbial communities in SS- and IGBS-inoculated MFCs. The results showed insignificant differences in bacterial richness and diversity between microbial communities of both MFCs, while SW differed significantly.

### 4.1. MFCs Treatment Efficiency of SW

Treatment of SW using MFCs inoculated with two different inoculums achieved substantial COD removal rates. A previous study found that a single-chambered MFC with a working volume 28 mL removed only 27% of the COD in SW having a high initial COD of 8,320 mg L^−1^ after 44 h (Min et al., 2005), whereas we found 76.4% and 65.7% removal of COD from SW by the SS-MFCs and IGBS-MFCs, respectively, after 48 h (data not shown). The average current density of the MFCs (Figure 1, Table 1) was within the range reported for other wastewater-fed MFC systems [5]. Consistent with other reports [13, 14], differences in external resistance within the range we tested (10–1000 *Ω*) did not notably alter the performance of the MFCs (data not shown).

Swine wastewater is characterized by high content of VFAs although their initial concentration in raw swine wastewater across different farms can vary substantially. Our results are consistent with others demonstrating that MFC treatment of SW largely eliminates VFAs, which are largely responsible for the SW odor [4]. Importantly, the SW feed in our experiments was approximately 5-fold higher strength and the HRT is less than five times that utilized by Jung et al. [4] and yet the MFCs still performed well at removing the VFAs.

In summary, use of SS as an anodic inoculum resulted in superior treatment performance of the MFCs over the 67 d course of the experiment compared to IGBS inoculum. This may indicate a more general tendency of preadapted inocula to perform better at degrading the substrate [15].

### 4.2. The Microbiome of Electrogenic of Anodic Biofilms and Planktonic Populations of MFCs

We used metagenomic analysis to explore the whole taxonomic diversity of the SS and IGBS inoculums, SW, and anodic and planktonic microbial communities of MFCs. Proteobacteria (mostly Deltaproteobacteria) became the predominant phylum in both MFCs anodic communities, while Firmicutes and Bacteroidetes decreased. The planktonic community of the IGBS-MFCs showed notable variation in relative abundance and became more similar to their anodic communities. In contrast, the planktonic communities of the SS-MFCs were intermediate between the SW and anodic communities. A previous study of a distillery wastewater-treating pilot-scale MFC inoculated with IGBS showed that the dominant anodic phyla (Proteobacteria, Bacteroidetes, and Firmicutes) were similar to that of the IGBS inoculum [16].

Previous studies have shown that SW could be used as a suitable inoculum for electricity production using MFCs, distinguished by the chamber and cathode types [4, 17]. Analysis of the anodic microbial communities in the SS-MFCs mainly showed that dominant species belonged to three major phyla Proteobacteria, Bacteroidetes, and Firmicutes [9, 12]. Results of metagenomics analysis in our study are in good agreement with results in the literature [9, 12].

Detailed analysis of the dominant anodic bacterial species in SW-treating MFCs showed high diversity in members of the Deltaproteobacteria, Gammaproteobacteria, Firmicutes*, Bacteroides,* and Archaea. Among all Deltaproteobacteria*, Geobacter metallireducens*,* Pelobacter propionicus*,* Desulfovibrio vulgaris, Syntrophobacter fumaroxidans,* and* Syntrophus aciditrophicus* were found to be the most abundant in the anodic microbial communities of both MFCs. The well-known electrogenic* Geobacter sulfurreducens*, dominant in the MFC microbial biofilms, generates a current via membrane c-type cytochromes* (omcZ)* and secretion of pili encoded by the* pilA* gene [3, 18, 19]. In contrast, anoditrophilic Fe(III)-reducing* Pelobacter carbinolicus* was characterized as a nonelectrogenic symbiotic bacterium responsible only for converting of substrates to acetate and hydrogen for use by* G. sulfurreducens* [3, 20]. Cytochrome *c* localized on the outer cell membrane of* Desulfovibrio desulfuricans* contributed to the electron transfer in an electricity-generating MFC [21].

Members of Deltaproteobacteria might contribute to VFA degradation. Our data demonstrate a relative abundance of* Syntrophobacter fumaroxidans* and* Syntrophus aciditrophicus* on the MFC anodes, which may aid in metabolism of propionic and butyric acid in the SW. Pure culture experiments with* Geobacter* species isolated from swine wastes examined the ability to biodegrade individual and mixtures of VFAs [22]. It was shown that* G. metallireducens, G. humireducens,* and* G. grbiciae* consume VFAs and stimulate VFAs oxidation depending on availability of Fe(III).

This study demonstrates that* Acinetobacter baumannii* and* Pseudomonas fluorescens* belonging to Gammaproteobacteria were prevalent members in the anodic community of both MFCs. The Gammaproteobacteria possess diverse metabolic capabilities involved in a breakdown of different substrates and production of soluble redox-active compounds, resulting in current generation in MFCs [3, 23, 24].* Acinetobacter* species dominating in the microbial community of MFCs fed with fermentable substrates were able to produce electricity [25]. Production of* pili*-like structures encoded by* csuC* and* csuE* genes in* A. baumannii* influences the colonization of different abiotic surfaces [26]. We found a considerable number of* Pseudomonas* species in both MFCs types. The ability of* Pseudomonas* to consume various carbon sources is known. Moreover, excretion of soluble electrochemically redox mediators participating in the electricity production in MFCs has been observed [23, 27]. Thus, dominant* Pseudomonas fluorescens* might be responsible for COD removal from the SW and the excretion of redox mediators contributes to the observed electricity generation of the MFCs.

The relatively high abundance of* Shewanella baltica* in the anodic microbial communities provides evidence of their importance in the conversion of COD into electricity. Previous studies have showed that electrogenic* Shewanella* species might transfer electrons to the anodes of MFCs either through nanowires or excretion of redox-active second metabolites [28, 29].

Our study demonstrates a relative abundance of bacteria related phyla Firmicutes and Bacteroidetes. It is well known that* Clostridium* species participate in fermentation processes and conversion of organic substrates to VFAs and hydrogen and that they are indigenous microbiota of the swine gastrointestinal tract and manure [12]. Bacteroidetes are widely recognized as the intestinal microflora associated with fermentation of carbohydrates and utilization of nitrogenous compounds, as well as odor production [30]. We found that the remaining dominant bacteria,* Flavobacterium johnsoniae* and* Bacteroides fragilis,* became even more abundant in the planktonic populations of both MFCs.

16S rRNA sequence analysis of a SW-treating MFC microbial community showed that two members of Firmicutes, a Gram-positive* Turicibacter* sp. and* Sedimentibacter* spp., were the dominant genera on the anodes of a MFC having a maximum power point tracking system [9]. Earlier studies demonstrated reduction of VFAs level depending on a seasonal shift of* Bacteroidetes* members in an anaerobic lagoon used for swine waste treatment [12].

In our study, two Archaea species* M. barkeri* and* M. thermautotrophicus* increased in the bacterial communities of SS-MFCs and IGBS-MFCs, respectively. Rotaru et al. established that the acetoclastic methanogen* M. barkeri* in association with electrogenic bacterium* G. metallireducens* participates in direct interspecies electron transfer (DIET) [31]. We found a potential for a DIET-type bacterial association between* M. barkeri* and* G. metallireducens* in the anodic microbial community of the SS-MFCs; possible association between* M. thermautotrophicus* and* G. metallireducens* was found in the anodic microbial community of IGBS-MFCs.

Taken together, the profiling of microbial community diversity based on similarity and phylogeny supports a model for development of electrogenic biofilm in MFCs from their inocula.

## 5. Conclusion

This research demonstrates the importance of inoculum source on the electrogenic and degradative activities and ultimate microbial community composition of SW-treating MFCs. MFC treatment of SW is a potentially more environmentally friendly alternative to energetically costly aerobic treatment or odorous space-demanding anaerobic lagoons. Particularly, the comprehensive analysis of SS- and IGBS-MFCs treated SW revealed that electricity production by MFC pairs remained relatively stable; however, the current density of the SS-MFCs (56.6 ± 2.4 mA m^−2^ 169) was higher. Both MFC pairs displayed the ammonia removal. Among all VFAs propionic and acetic acids were found as dominated. Negligible concentrations indoles were detected. Aromatic compounds as p-cresol and phenol were not found. Analysis of microbial communities of both MFCs showed that MFC anodic communities form their own distinct cluster which contains* Geobacter* spp., represented by eight predominant and three minor species in both MFC anodic communities. Clustering of microbial communities based on dominant bacterial genera indicates that the electrogenic communities in the MFC developed from their inocula. Spectrum of dominated bacteria is significantly enriched by genera* Pelobacter, Pseudomonas, Arcobacter, Syntrophus, Syntrophobacter, Bacteroides, and Clostridium* and two acetoclastic methanogens (*Methanosarcina* and* Methanothermobacter*).

## Supplementary Material

Figure S1: Cell voltage and power density vs. current density (cell polarization) of MFCs (A) inoculated with swine sludge (SS); (B) inoculated with industrial granular brewery sludge (IGSB). Open circles, voltage; closed circles, power density.
Figure S2: Current generation during swine wastewater treatment by MFCs inoculated with swine waste sludge and brewery sludge. Mean data from duplicate experiments; error bars indicating ±SD to not exceed the diameter of the data point symbols. Open boxes, SS-inoculated MFC; open diamonds, IGBS-inoculated MFC.
Figure S3: Total COD concentrations in SW feed and within MFCs inoculated with swine waste sludge and industrial granular brewery sludge. Squares, SS-inoculated MFC; circles, IGBS-inoculated MFC; Mean data from duplicate experiments.
Figure S4: Change in total phosphorus (PO_4_^3^^−^-P) in inflow and outflows of MFCs inoculated with swine wastewater sludge (SS) and industrial granular brewery sludge (IGBS). Mean data from duplicate experiments with error bars (±SD).
Figure S5: Changes in ammonia nitrogen (NH_4_-N) in inflow and outflows of MFCs inoculated with swine wastewater sludge (SS) and industrial granular brewery sludge (IGBS). Mean data from duplicate experiments with error bars (±SD).
Figure S6: SEM images of the anodic biofilms the MFCs inoculated with (A) swine waste and (B) brewery sludge. Samples of anode surfaces (activated carbon granules and fiber) the MFCs were taken after 67 days of swine wastewater treatment upon disassembling the MFCs. Slices of anode electrodes (1 cm^2^) were briefly rinsed with deionized water and fixed in 2.5% glutaraldehyde for 2 h, further in 1% osmium tetroxide. Dehydration of microbial biofilms was carried out using a series of ethanol–water solutions (25, 50, 75, 95, 100%). After gold coating, the obtained specimens were observed using a Focused Ion Beam Scanning electron microscope (Helios NanoLab 650, USA). High resolution images were acquired using an accelerating voltage of 20 kV at a working distance of 3.1–6.5 mm.
Table S1: Summary of MFC operation modes.
Table S2: VFA concentrations in SS-inoculated MFCs.
Table S3: VFA concentrations in IGBS-inoculated MFCs.
Table S4: Diversity of dominated species in the SW, inocula, anodic and planktonic microbial communities of SS- and IGBS-inoculated MFCs.

## Figures and Tables

**Table 1:** Treatment-related characteristics of swine wastewater-fed microbial fuel cells.

Source	COD (mg L^−1^)^[a,b]^	VFA (mg L^−1^)^[a,b]^	NH_4_^+^-N (mg L^−1^)^[a,b]^	PO_4_^3−^-P (mg L^−1^)^[a,b]^
SW inflow	6824	1452	365	374
SS-inoculated MFC	1684 (−75.3%)	222 (−84.8%)	286 (−21.6%)	365 (−2.4%)
IGBS-inoculated MFC	2219 (−67.5%)	314 (−78.4%)	327 (−10.4%)	370 (−1.1%)

^[a]^Results are means of measurements taken of two independently operating MFCs for both MFC types, both operating with 100 Ω external resistance sampled at 67 days following initiation of operations. ^[b]^Percent change in parentheses.

**Table 2:** Removal of selected volatile fatty acids by swine wastewater-fed microbial fuel cells.

Source	Concentration (mg L^−1^)^*∗*^						
	Acetate	Propionate	Isobutyrate	Butyrate	Isovalerate	Valerate	Hexanoate
SW inflow	114.95	425.26	10.40	198.07	81.48	127.26	96.56
SS-inoculated MFC effluent	7.06 ± 3.65	59.98 ± 51.37	0.90 ± 0.78	0.80 ± 0.46	5.62 ± 4.79	0.98 ± 0.88	0.51 ± 0.29
IGBS- inoculated MFC effluent	8.78 ± 3.72	108.65 ± 25.54	6.24 ± 1.60	4.50 ± 1.12	40.89 ± 7.41	12.26 ± 3.90	4.87 ± 0.83

^*∗*^±Range of variation between the two MFCs of each type.
