# Supplementary material for: Comparative Metagenomic Analysis of Electrogenic Microbial Communities in Differentially Inoculated Swine Wastewater-Fed Microbial Fuel Cells
Source: Scientifica (Cairo). 2017 Oct 12;2017:7616359. doi: 10.1155/2017/7616359 (PMC5660801; doi:10.1155/2017/7616359)
Supplement: Supplementary file 1 — Figure S1: Cell voltage and power density vs. current density (cell polarization) of MFCs (A) inoculated with swine sludge (SS); (B) inoculated with industrial granular brewery sludge (IGSB). Open circles, voltage; closed circles, power density. Figure S2: Current generation during swine wastewater treatment by MFCs inoculated with swine waste sludge and brewery sludge. Mean data from duplicate experiments; error bars indicating ±SD to not exceed the diameter of the data point symbols. Open boxes, SS-inoculated MFC; open diamonds, IGBS-inoculated MFC. Figure S3: Total COD concentrations in SW feed and within MFCs inoculated with swine waste sludge and industrial granular brewery sludge. Squares, SS-inoculated MFC; circles, IGBS-inoculated MFC; Mean data from duplicate experiments. Figure S4: Change in total phosphorus (PO43−-P) in inflow and outflows of MFCs inoculated with swine wastewater sludge (SS) and industrial granular brewery sludge (IGBS). Mean data from duplicate experiments with error bars (±SD). Figure S5: Changes in ammonia nitrogen (NH4-N) in inflow and outflows of MFCs inoculated with swine wastewater sludge (SS) and industrial granular brewery sludge (IGBS). Mean data from duplicate experiments with error bars (±SD). Figure S6: SEM images of the anodic biofilms the MFCs inoculated with (A) swine waste and (B) brewery sludge. Samples of anode surfaces (activated carbon granules and fiber) the MFCs were taken after 67 days of swine wastewater treatment upon disassembling the MFCs. Slices of anode electrodes (1 cm2) were briefly rinsed with deionized water and fixed in 2.5% glutaraldehyde for 2 h, further in 1% osmium tetroxide. Dehydration of microbial biofilms was carried out using a series of ethanol–water solutions (25, 50, 75, 95, 100%). After gold coating, the obtained specimens were observed using a Focused Ion Beam Scanning electron microscope (Helios NanoLab 650, USA). High resolution images were acquired using an accelerating voltage of 20 kV [file 7616359.f1.pdf]

Supplementary materials

Comparative metagenomic analysis of electrogenic microbial communities in differentially inoculated swine wastewater-fed microbial fuel cells

Irina V. Khilyas[1*], Anatoly A. Sorokin[3,4], Larisa Kiseleva[2], David J.W. Simpson[2], V. Fedorovich[2], Margarita R. Sharipova[1], Mami Kainuma[2], Michael F. Cohen[2,5], and Igor Goryanin[2,6,7]

[1]Institute of fundamental medicine and biology, Kazan (Volga Region) Federal University, Kazan, Russian Federation

[2]Biological Systems Unit, Okinawa Institute of Science and Technology, Okinawa, Japan

[3]Institute of Cell Biophysics Russian Academy of Science, Pushcino, Moscow region, Russian Federation

[4]Moscow Institute of Physics and Technology, Dolgoprudnyi, Moscow Region, Russian Federation

[5]Department of Biology, Sonoma State University, Rohnert Park, CA, USA

[6]School of Informatics, University of Edinburgh, Edinburgh, United Kingdom

[7] Tianjin Institute of Industrial Biotechnology, China

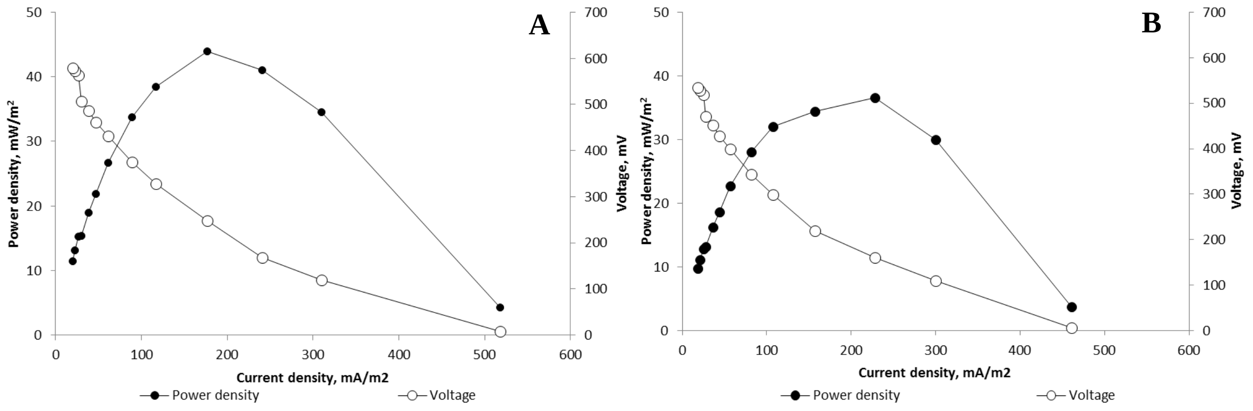

Fig. S1. Cell voltage and power density vs. current density (cell polarization) of MFCs (A) inoculated with swine sludge (SS); (B) inoculated with industrial granular brewery sludge (IGSB). Open circles, voltage; closed circles, power density.

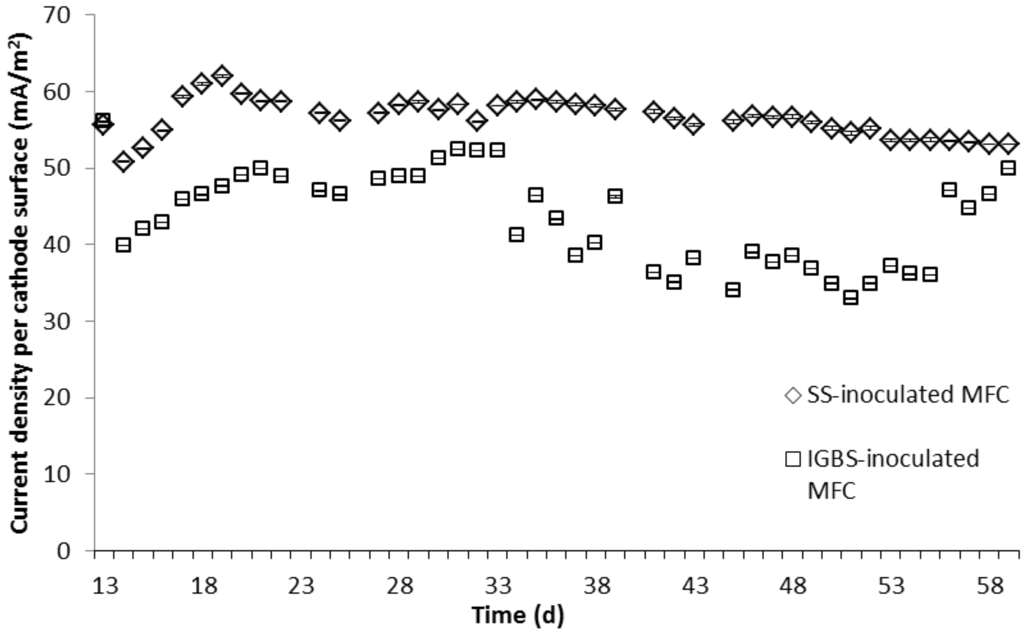

Fig. S2. Current generation during swine wastewater treatment by MFCs inoculated with swine waste sludge and brewery sludge. Mean data from duplicate experiments; error bars indicating ±SD to not exceed the diameter of the data point symbols. Open boxes, SS-inoculated MFC; open diamonds, IGBS-inoculated MFC.

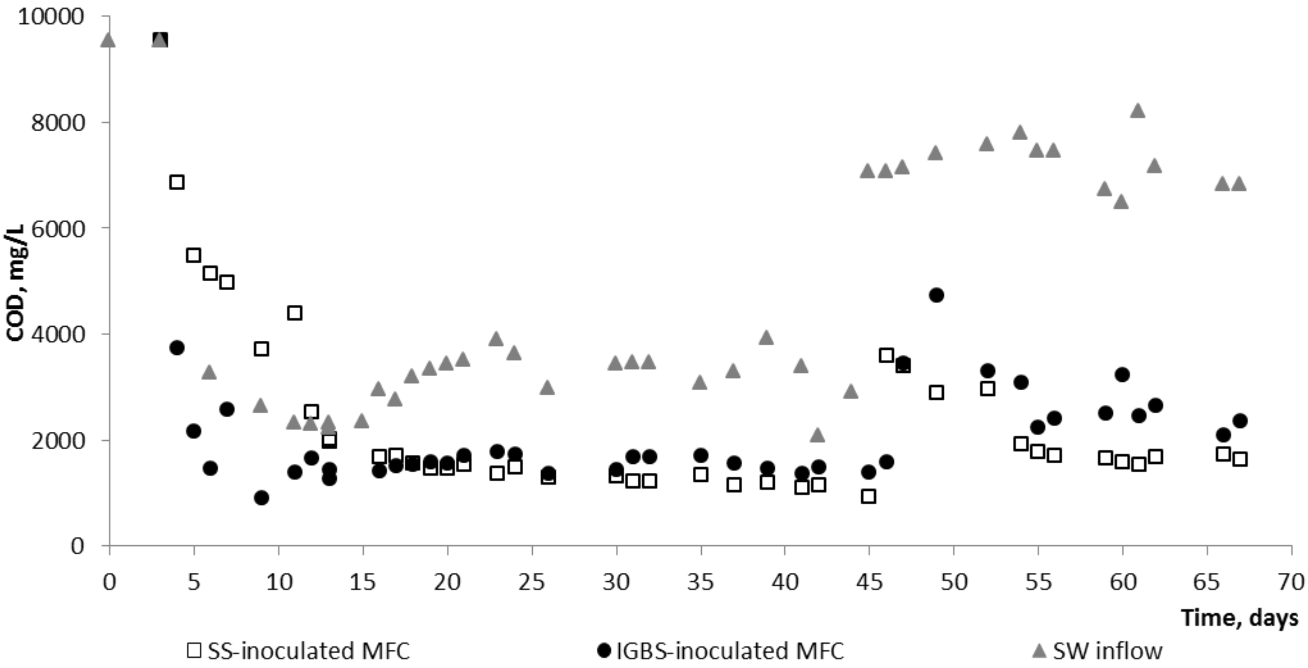

Fig. S3. Total COD concentrations in SW feed and within MFCs inoculated with swine waste sludge and industrial granular brewery sludge. Squares, SS-inoculated MFC; circles, IGBS-inoculated MFC; Mean data from duplicate experiments.

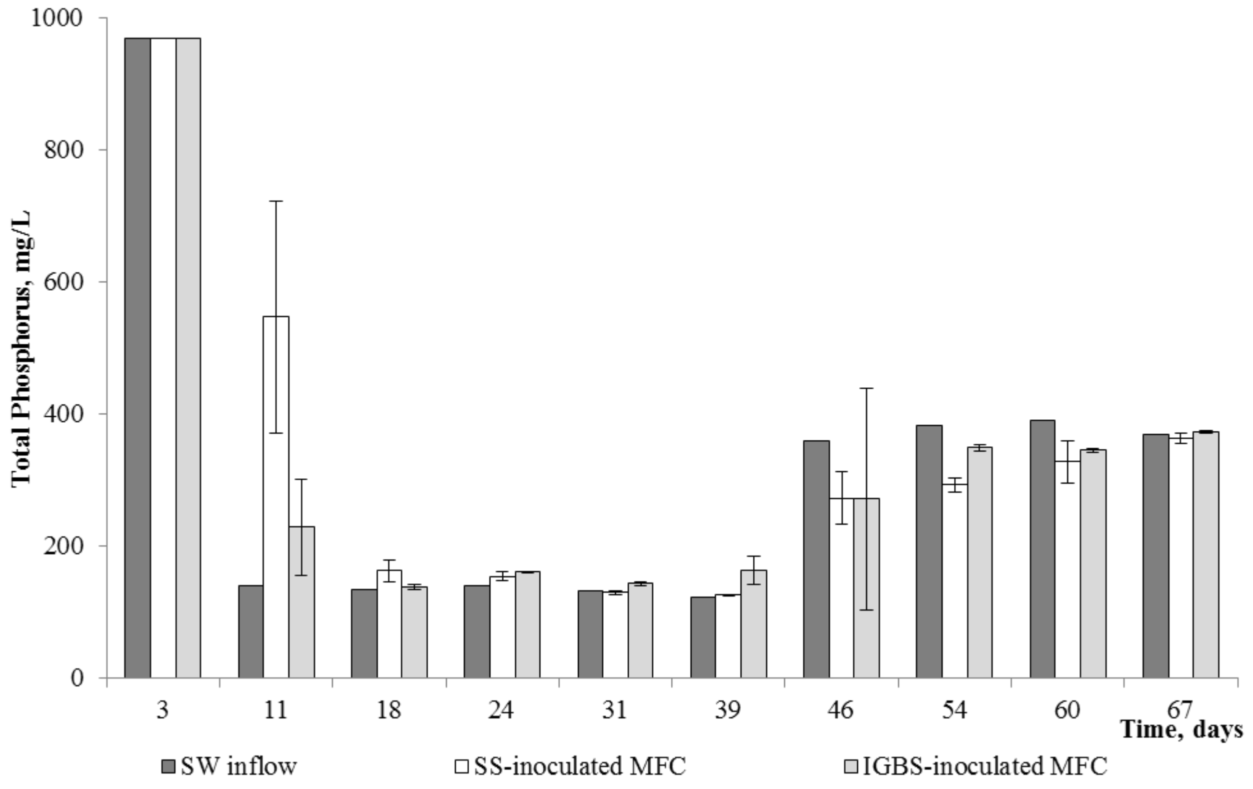

Fig. S4. Change in total phosphorus (PO_4^{3-}-P) in inflow and outflows of MFCs inoculated with swine wastewater sludge (SS) and industrial granular brewery sludge (IGBS). Mean data from duplicate experiments with error bars (±SD).

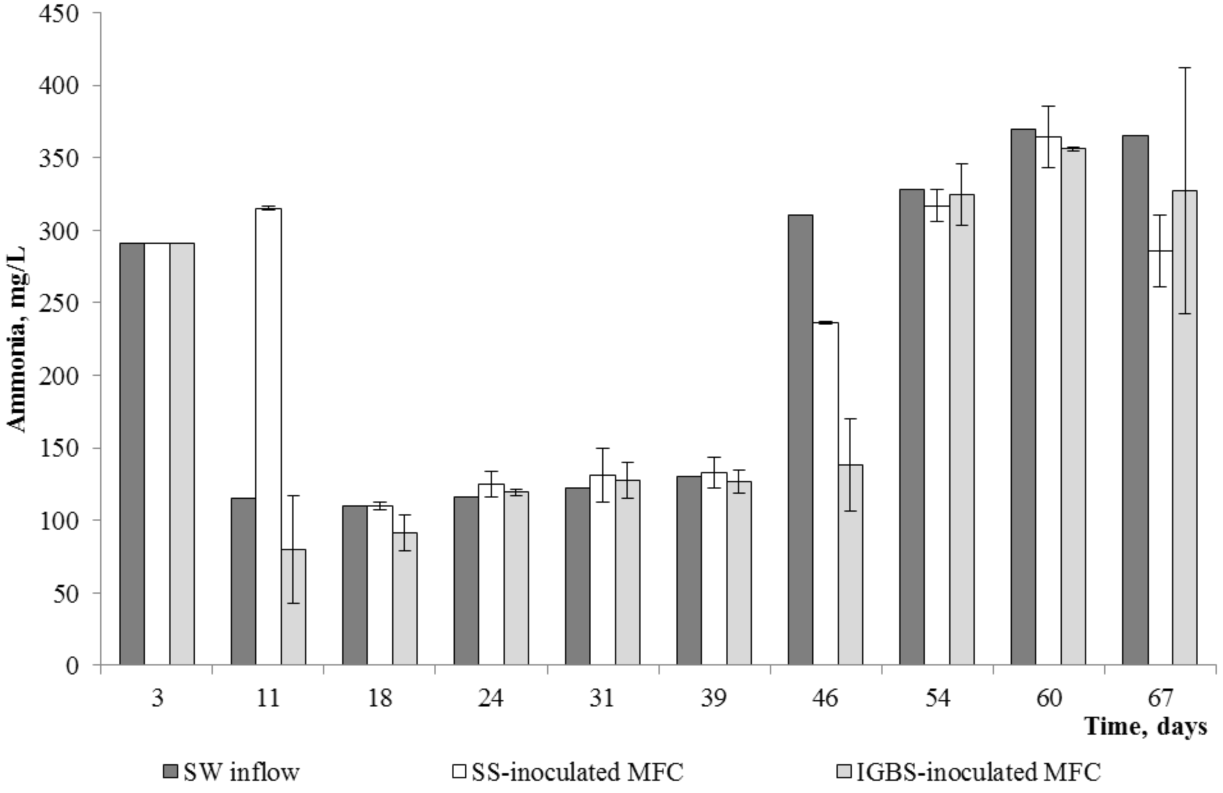

Fig. S5. Changes in ammonia nitrogen (NH_4-N) in inflow and outflows of MFCs inoculated with swine wastewater sludge (SS) and industrial granular brewery sludge (IGBS). Mean data from duplicate experiments with error bars (±SD).

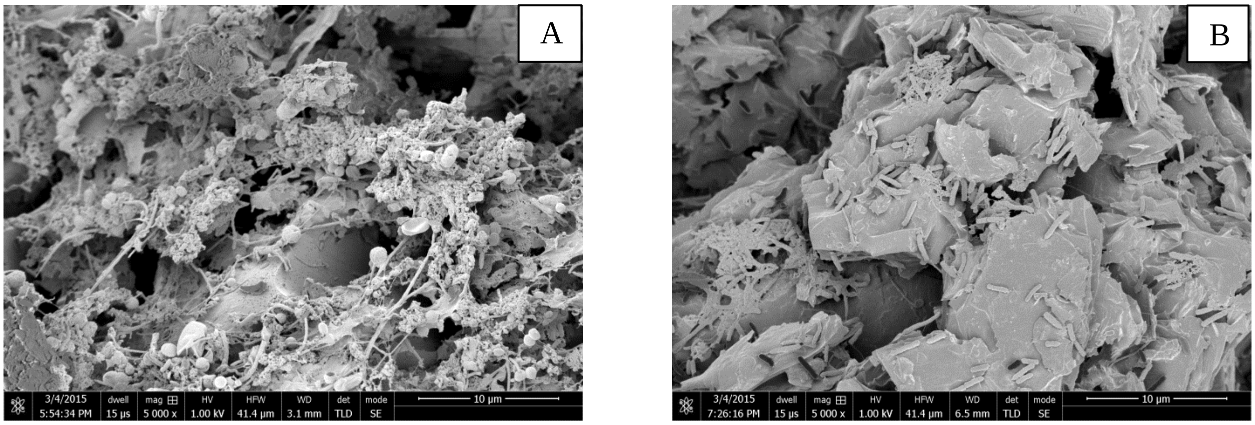

Fig. S6. SEM images of the anodic biofilms the MFCs inoculated with (A) swine waste and (B) brewery sludge. Samples of anode surfaces (activated carbon granules and fiber) the MFCs were taken after 67 days of swine wastewater treatment upon disassembling the MFCs. Slices of anode electrodes (1 cm^2) were briefly rinsed with deionized water and fixed in 2.5% glutaraldehyde for 2 h, further in 1% osmium tetroxide. Dehydration of microbial biofilms was carried out using a series of ethanol–water solutions (25, 50, 75, 95, 100%). After gold coating, the obtained specimens were observed using a Focused Ion Beam Scanning electron microscope (Helios NanoLab 650, USA). High resolution images were acquired using an accelerating voltage of 20 kV at a working distance of 3.1–6.5 mm.

Table S1. Summary of MFC operation modes.

Step	Period, days	Feeding mode	HRT, h	Dilution rate, times	Initial pH	External resistance	Description
I	0-3	Inoculation of units	-	-	6.8	Open circuit	Biofilm formation
II	3-12	Every 2 days	48	-	6.8	Open circuit	Biofilm adaptation
III	13-35	every 2 h, during 2 min 34 sec, V=180mL/day	24	6	6.8	Closed circuit (1000 Ω)	Treatment of wastes
IV	36-41	every 2 h, during 2 min 34 sec, V=180mL/day	24	6	5.5	Closed circuit (1000 Ω)	Influence of initial pH on treatment processes
V	42-44	every 2 h, during 2 min 34 sec, V=180mL/day	24	6	6.8	Closed circuit (1000 Ω)	Preparing to mode changing
VI	45-52	every 2 h, during 2 min 34 sec, V=180mL/day	24	2	6.8	Closed circuit (1000 Ω)	Increasing of initial COD level
VII	53-59	every 2 h, during 1 min 34 sec, V=90mL/day	48	2	6.8	Closed circuit (1000 Ω)	Optimization of MFC wastes treatment processes
VIII	60-62	every 2 h, during 1 min 34 sec, V=90mL/day	48	2	6.8	closed circuit (10 Ω)	Influence of low external resistance on MFC wastes treatment
IX	63-64	every 2 h, during 1 min 34 sec, V=90mL/day	48	2	6.8	From 0 – 2100 Ω	Optimization of external resistance on MFC wastes treatment
X	65-66	every 2 h, during 1 min 34 sec, V=90mL/day	48	2	6.8	closed circuit (100 Ω)	Influence of middle external resistance on MFC wastes treatment
XI	67	The End of MFC treatment					

Table S2. VFA concentrations in SS-inoculated MFCs.

	Concentration, mg/L						
Time, days	Acetate	Propionate	Isobutyrate	Butyrate	Isovalerate	Valerate	Hexanoate
13	33.73 (±2.08)	83.44 (±4.37)	4.10 (±0.37)	30.27 (±3.83)	21.16 (±1.66)	17.51 (±2.10)	11.99 (±1.88)
18	20.04 (±3.0)	84.70 (±8.31)	6.44 (±0.53)	36.34 (±11.04)	34.49 (±0.77)	38.34 (±0.79)	20.43 (±4.52)
24	11.63 (±2.26)	55.00 (±20.76)	4.11 (±1.59)	19.81 (±14.03)	22.16 (±10.57)	16.39 (±8.05)	9.59 (±5.29)
31	6.65 (±1.01)	47.95 (±6.19)	4.21 (±0.96)	9.47 (±6.62)	21.46 (±7.66)	13.87 (±6.01)	7.46 (±4.57)
39	10.61 (±2.46)	55.36 (±15.37)	3.30 (±1.36)	4.27 (±2.29)	13.75 (±8.09)	8.95 (±5.61)	4.30 (±3.27)
46	45.79 (±3.77)	202.24 (±19.87)	8.26 (±0.85)	67.45 (±14.50)	49.09 (±5.37)	60.49 (±8.88)	40.22 (±9.13)
52	9.70 (±3.09)	105.76 (±23.07)	5.53 (±1.18)	3.27 (±1.85)	63.22 (±1.92)	11.93 (±6.66)	7.78 (±2.37)
60	9.43 (±1.28)	38.01 (±13.65)	0.90 (±0.49)	4.91 (±0.45)	5.39 (±2.11)	3.12 (±0.14)	2.64 (±0.01)
67	7.06 (±3.65)	59.98 (±51.37)	0.90 (±0.78)	0.80 (±0.46)	5.62 (±4.79)	0.98 (±0.88)	0.51 (±0.29)

* (±) Range of variation represent duplicate experiments.

Table S3. VFA concentrations in IGBS-inoculated MFCs.

	Concentration, mg/L						
Time, days	Acetate	Propionate	Isobutyrate	Butyrate	Isovalerate	Valerate	Hexanoate
13	18.22 (±1.54)	27.44 (±6.99)	1.94 (±0.39)	12.50 (±2.03)	6.65 (±0.62)	7.92 (±0.07)	6.52 (±0.06)
18	26.14 (±6.64)	69.00 (±31.26)	4.44 (±0.37)	33.95 (±13.52)	23.06 (±0.78)	25.57 (±4.79)	20.80 (±3.00)
24	31.67 (±3.00)	68.73 (±2.64)	5.15 (±0.76)	30.44 (±1.19)	27.37 (±4.89)	20.64 (±0.89)	15.32 (±0.44)
31	16.86 (±2.72)	47.74 (±14.49)	4.19 (±0.58)	17.47 (±6.65)	26.34 (±3.80)	17.43 (±5.81)	13.35 (±5.01)
39	14.04 (±0.90)	45.07 (±3.85)	3.29 (±0.03)	12.09 (±2.08)	18.30 (±0.59)	11.04 (±1.10)	7.03 (±0.51)
46	16.14 (±3.36)	48.94 (±11.57)	3.09 (±0.36)	12.85 (±3.71)	20.28 (±2.44)	12.50 (±3.59)	8.26 (±2.08)
52	15.91 (±2.02)	85.48 (±19.12)	7.02 (±1.64)	17.50 (±2.56)	56.10 (±11.26)	21.02 (±3.93)	14.08 (±3.56)
60	15.05 (±1.45)	77.66 (±6.39)	5.48 (±0.24)	10.10 (±2.47)	43.23 (±0.30)	12.70 (±3.29)	6.76 (±1.63)
67	8.78 (±3.72)	108.65 (±25.54)	6.24 (±1.60)	4.50 (±1.12)	40.89 (±7.41)	12.26 (±3.90)	4.87 (±0.83)

* (±) Range of variation represent duplicate experiments.

Table S4. Diversity of dominated species in the SW, inocula, anodic and planktonic microbial communities of SS- and IGBS-inoculated MFCs.

Total hit numbers of class ***Deltaproteobacteria***

	Swine wastewater	SS-inoculated MFC, Anode	SS-inoculated MFC, Planktonic	IGBS-inoculated MFC, Anode	IGBS-inoculated MFC, Planktonic	IGBS	Swine sludge
Deltaproteobacteria	28255	318508.75	70918.25	487004.25	142937.5	149374	44033.5

Prokaryotes abundance, ***Geobacter*** species (17 OTUs)

	Swine wastewater	SS-inoculated MFC, Anode	SS-inoculated MFC, Planktonic	IGBS-inoculated MFC, Anode	IGBS-inoculated MFC, Planktonic	IGBS	Swine sludge
Geobacter metallireducens	824	106093	2309	115774	4840	5240	1152
Geobacter sulfurreducens	853	43073	2262	80771	5860	6654	1308
Geobacter uraniireducens	1008	14489	2571	36737	6470	7026	1497
Geobacter lovleyi	551	7149	1416	31628	3062	2152	680
Geobacter bemidjiensis	495	4420	1140	12923	2543	2753	662
Geobacter sp. FRC-32	267	4272	895	11054	2221	2458	509
Geobacter sp. M18	140	4092	810	9996	2037	2251	378
Geobacter sp. M21	216	2606	485	6313	1082	1225	292
uncultured *Geobacter* sp.	1	16	8	141	14	13	1
Geobacter sp.	0	14	11	48	10	10	4
Geobacter psychrophilus	0	0	0	13	0	0	0
Geobacter grbiciae	0	6	0	6	0	0	0
Geobacter chapelleii	0	0	0	11	0	0	0
Geobacter thiogenes	0	0	0	3	0	0	0
Geobacter sp. Ply1	0	0	0	3	0	0	0
Geobacter pelophilus	0	0	0	1	0	0	0
Geobacter sp. Ply4	0	0	0	0	0	0	0

Prokaryotes abundance, ***Pelobacter*** species (5 OTUs)

	Swine wastewater	SS-inoculated MFC, Anode	SS-inoculated MFC, Planktonic	IGBS-inoculated MFC, Anode	IGBS-inoculated MFC, Planktonic	IGBS	Swine sludge
Pelobacter propionicus	1227	12472	2919	65096	6367	6325	1605
Pelobacter carbinolicus	987	6022	1965	4919	3596	4013	1186
Pelobacter acidigallici	1	2	2	4	7	14	2
Pelobacter acetylenicus	0	0	0	2	2	3	0
Pelobacter massiliensis	0	0	0	0	1	1	0

Prokaryotes abundance, ***Desulfovibrio*** species (18 OTUs)

	Swine wastewater	SS-inoculated MFC, Anode	SS-inoculated MFC, Planktonic	IGBS-inoculated MFC, Anode	IGBS-inoculated MFC, Planktonic	IGBS	Swine sludge
Desulfovibrio vulgaris	2352	7150	2309	6780	3797	3134	2945
Desulfovibrio desulfuricans	2313	3910	1282	5324	2042	967	3825
Desulfovibrio alaskensis	1094	3870	1495	3510	2594	2556	1359
Desulfovibrio magneticus	462	3831	1089	4067	1803	1581	619
Desulfovibrio sp. FW1012B	223	2805	679	2882	1137	1009	371
Desulfovibrio fructosovorans	203	2539	707	2492	1186	939	326
Desulfovibrio sp. 3_1_syn3	983	1181	465	1784	748	302	1566
Desulfovibrio aespoeensis	215	2393	625	1368	943	937	287
Desulfovibrio salexigens	397	1635	695	1133	957	807	390
Desulfovibrio piger	975	667	335	790	404	285	1129
Desulfovibrio gigas	11	67	28	79	64	62	19
Desulfovibrio sp.	2	10	6	5	3	3	3
Desulfovibrio africanus	3	4	1	1	0	0	0
Desulfovibrio butyratiphilus	0	0	0	1	0	0	0
Desulfovibrio sp. JD160	0	1	0	0	0	0	0
Desulfovibrio sp. LG-2009	0	0	0	1	0	0	0
Desulfovibrio burkinensis	0	0	0	0	0	0	0
Desulfovibrio sulfodismutans	0	0	0	0	0	0	0

Prokaryotes abundance, ***Syntrophus*** species (1 OTUs)

	Swine wastewater	SS-inoculated MFC, Anode	SS-inoculated MFC, Planktonic	IGBS-inoculated MFC, Anode	IGBS-inoculated MFC, Planktonic	IGBS	Swine sludge
Syntrophus aciditrophicus	1038	11935	11550	10281	19335	27383	5232

Prokaryotes abundance, ***Syntrophobacter*** species (2 OTUs)

	Swine wastewater	SS-inoculated MFC, Anode	SS-inoculated MFC, Planktonic	IGBS-inoculated MFC, Anode	IGBS-inoculated MFC, Planktonic	IGBS	Swine sludge
Syntrophobacter fumaroxidans	958	8255	4860	18654	20536	17987	2615
Syntrophobacter pfennigii	0	0	0	0	0	0	0

Total hit numbers of class ***Gammaproteobacteria***

	Swine wastewater	SS-inoculated MFC, Anode	SS-inoculated MFC, Planktonic	IGBS-inoculated MFC, Anode	IGBS-inoculated MFC, Planktonic	IGBS	Swine sludge
Gammaproteobacteria	513175.5	97953	49742	131568.75	142295.75	213992	506025.5

Prokaryotes abundance, ***Acinetobacter*** species (56 OTUs)

	Swine wastewater	SS-inoculated MFC, Anode	SS-inoculated MFC, Planktonic	IGBS-inoculated MFC, Anode	IGBS-inoculated MFC, Planktonic	IGBS	Swine sludge
Acinetobacter baumannii	38509	5443	3970	3326	5368	309	14869
Acinetobacter sp. ADP1	17655	2505	1795	1698	2532	167	7012
Acinetobacter johnsonii	16163	2146	1596	1073	1831	42	6034
Acinetobacter lwoffii	15299	1874	1364	1018	1571	56	5778
Acinetobacter oleivorans	6340	810	587	447	815	42	2431
Acinetobacter radioresistens	5024	722	536	477	695	42	1923
Acinetobacter junii	3322	620	407	333	718	29	1300
Acinetobacter haemolyticus	2449	413	261	229	457	26	932
Acinetobacter calcoaceticus	1583	223	158	146	246	28	646
Acinetobacter sp. ATCC 27244	1556	249	183	143	305	14	568
Acinetobacter pittii	1509	204	160	148	191	16	589
Acinetobacter nosocomialis	1174	196	119	144	187	14	469
Acinetobacter venetianus	165	18	13	8	27	4	75
Acinetobacter sp.	59	11	7	6	10	0	18
Acinetobacter sp. LUH5605	42	7	6	2	7	0	16
Acinetobacter sp. P8-3-8	27	4	5	2	5	0	12
Acinetobacter schindleri	28	1	0	0	3	0	12
Acinetobacter guillouiae	26	2	2	2	2	0	7
Acinetobacter sp. SUN	26	1	1	2	2	0	8
Acinetobacter sp. NFM2	22	2	2	4	3	0	2
Acinetobacter sp. SE19	12	5	1	3	6	0	5
Acinetobacter sp. M-1	15	1	0	0	4	0	3
Acinetobacter sp. XMZ-26	16	0	0	0	0	0	8
Acinetobacter sp. MO	9	0	0	0	0	0	6
Acinetobacter baylyi	6	0	0	0	2	0	4
Acinetobacter sp. BD413	7	1	0	0	1	0	3
Acinetobacter towneri	8	0	0	0	0	0	4
Acinetobacter sp. CR1	9	0	0	0	0	0	3
Acinetobacter bereziniae	8	0	0	0	0	0	3
Acinetobacter sp. AD513A	3	3	1	0	1	0	2
Acinetobacter bouvetii	4	1	0	0	1	0	3
Acinetobacter sp. no. 6	4	1	1	0	0	0	1
Acinetobacter sp. 1690	5	0	0	0	0	0	3
Acinetobacter genomo sp. 13	4	0	0	1	0	0	2
Acinetobacter sp. NCIMB9871	2	0	0	0	1	0	2
Acinetobacter tandoii	6	0	0	0	0	0	0
Acinetobacter sp. DM-2005	4	0	0	0	0	0	2
Acinetobacter sp. G16(2009)	4	0	0	0	0	0	2
Acinetobacter sp. 511B	2	0	0	0	0	0	1
Acinetobacter sp. 20	1	1	0	0	0	0	1
Acinetobacter sp. 423D	3	0	0	0	0	0	0

Acinetobacter sp. HY-7	3	0	0	0	0	0	1
Acinetobacter sp. A3-6	2	0	0	0	0	0	1
Acinetobacter genomosp. 6	2	0	0	0	0	0	1
Acinetobacter ursingii	1	0	0	0	0	0	0
Acinetobacter sp. TS25	0	1	0	0	0	0	0
Acinetobacter sp. UA3	1	0	0	0	0	0	2
Acinetobacter genomosp. 15TU	0	0	0	0	1	0	0
Acinetobacter glutaminasificans	0	0	0	0	0	0	1
Acinetobacter sp. AD321	0	0	0	0	0	0	0
Acinetobacter sp. RAG-1	0	0	0	0	0	0	0
Acinetobacter sp. 48A1	1	0	0	0	0	0	0
Acinetobacter sp. 8637	1	0	0	0	0	0	0
Acinetobacter sp. PD12	1	0	0	0	0	0	0
Acinetobacter sp. isolate 30Bi	0	0	0	0	0	0	0

Prokaryotes abundance, ***Pseudomonas*** species (119 OTUs)

	Swine wastewater	SS-inoculated MFC, Anode	SS-inoculated MFC, Planktonic	IGBS-inoculated MFC, Anode	IGBS-inoculated MFC, Planktonic	IGBS	Swine sludge
Pseudomonas fluorescens	4055	1893	1109	17643	3953	13719	2337
Pseudomonas aeruginosa	5319	2863	868	4520	5423	1488	3056
Pseudomonas putida	3294	1430	547	3179	2861	1302	1882
Pseudomonas mendocina	4051	1203	450	1774	3143	513	1997
Pseudomonas stutzeri	3824	1482	525	2001	2667	497	1974
Pseudomonas entomophila	1005	528	189	1105	973	416	562
Pseudomonas syringae group genomosp. 3	1052	440	191	1063	743	627	656
Pseudomonas syringae	769	424	180	1173	719	470	482
Pseudomonas savastanoi	820	434	190	952	789	500	482
Pseudomonas amygdali	252	99	37	199	168	122	154
Pseudomonas coronafaciens	117	62	18	104	86	62	59
Pseudomonas alcaligenes	21	26	8	27	52	8	9
Pseudomonas resinovorans	9	23	7	30	39	7	15
Pseudomonas alkylphenolia	4	8	2	36	43	6	3
Pseudomonas veronii	0	3	2	28	21	12	0
Pseudomonas chlororaphis	4	6	1	20	8	6	4
Pseudomonas denitrificans	4	12	2	26	5	0	1
Pseudomonas nitroreducens	16	4	0	8	10	3	7
Pseudomonas sp. TJI-51	11	2	1	7	11	2	3
Pseudomonas sp. MIS38	0	0	0	27	2	0	0
Pseudomonas anguilliseptica	0	7	1	14	2	0	1
Pseudomonas pseudoalcaligenes	9	2	0	4	6	1	2
Pseudomonas knackmussii	1	1	1	5	5	9	1
Pseudomonas brassicacearum	4	1	2	6	0	4	3
Pseudomonas oleovorans	9	1	1	3	2	0	1
Pseudomonas sp. CT14	1	8	1	4	3	0	0
Pseudomonas sp. Y2	3	1	0	4	4	0	4
Pseudomonas sp. MT-1	12	0	0	0	3	0	1
Pseudomonas sp. Chol1	0	5	0	4	5	0	1
Pseudomonas fragi	4	2	0	2	2	1	2
Pseudomonas sp. J465	3	0	0	0	4	3	2
Pseudomonas sp. S-47	2	0	0	6	0	3	1
Pseudomonas sp. JR1	1	0	1	3	2	2	1
Pseudomonas tolaasii	0	1	1	2	2	3	2
Flavobacterium lutescens	3	3	0	3	2	0	0
Pseudomonas marginalis	0	0	0	5	0	6	0
Pseudomonas abietaniphila	0	1	0	5	4	0	0

Pseudomonas extremaustralis	1	1	0	3	1	2	0
Pseudomonas sp. HR199	2	1	0	1	3	1	1
Pseudomonas viridiflava	2	0	0	1	1	1	3
Pseudomonas citronellolis	2	1	1	1	2	2	1
Pseudomonas sp. ND6	1	0	0	4	2	1	0
Pseudomonas sp. WBC-3	0	2	4	1	1	0	0
Pseudomonas sp. 2663	0	2	0	3	2	0	0
Pseudomonas chloritidismutans	5	0	0	0	0	0	3
Pseudomonas cichorii	0	1	0	0	0	3	1
Pseudomonas sp. NCIMB 10558	0	1	0	4	0	1	0
Pseudomonas sp. M1	0	0	0	2	1	1	1
Pseudomonas sp. 1-7	0	1	0	1	2	1	0
Pseudomonas sp. OM2164	0	1	0	1	1	0	1
Pseudomonas corrugata	3	0	0	0	0	0	0
Pseudomonas sp. WO24	0	2	0	1	2	0	0
Pseudomonas sp. CG21	0	0	0	0	1	1	1
Pseudomonas mediterranea	0	0	0	0	0	0	1
Pseudomonas sp. PCL1171	0	0	0	2	0	0	0
Pseudomonas otitidis	1	0	0	0	0	0	1
Pseudomonas fulva	1	0	0	1	0	0	0
Pseudomonas sp. OPS1	0	1	0	0	1	0	0
Pseudomonas sp. UK4	0	0	0	2	0	1	0
Pseudomonas sp. 7197	1	0	0	0	0	0	0
Pseudomonas sp. JS189	1	0	0	0	0	0	0
Pseudomonas sp. NyZ402	0	0	0	0	0	0	0
Pseudomonas sp. PC17	1	0	0	0	0	0	2
Pseudomonas sp. SLT2001	1	0	0	1	0	0	0
Pseudomonas sp. TW3	0	1	1	0	0	0	0
Pseudomonas synxantha	0	0	0	1	0	0	0
Pseudomonas alcaliphila	1	0	0	0	0	0	1
Pseudomonas sp. 61-3	0	0	0	0	1	1	0
Pseudomonas sp. TS44	0	0	0	0	1	0	0
Pseudomonas fuscovaginae	0	0	0	1	0	0	0
Pseudomonas psychrophila	0	0	0	0	0	0	1
Pseudomonas sp. B-0831	0	0	0	1	0	0	0
Pseudomonas sp. In5	1	0	0	0	0	0	1
Pseudomonas tuomuerensis	2	0	0	0	0	0	0
Pseudomonas frederiksbergensis	0	0	0	1	0	0	0
Pseudomonas sp. CT07	0	0	0	1	0	1	0
Pseudomonas sp. S11	1	0	0	0	0	0	0
Pseudomonas sp. XJUHX-1	0	0	0	1	0	0	0
Pseudomonas diterpeniphila	0	0	0	1	0	0	0
Pseudomonas reinekei	0	0	0	1	0	0	0
Pseudomonas sp. 122	0	0	0	0	0	1	0
Pseudomonas sp. Tik3	0	0	0	0	0	0	0
Pseudomonas azotoformans	0	0	0	0	0	1	0
Pseudomonas grimontii	0	0	0	0	0	0	0
Pseudomonas hydrogenovora	0	0	0	0	0	0	0
Pseudomonas qianpuensis	1	0	0	0	0	0	0
Pseudomonas sp. LQ26	0	0	0	0	1	0	0
Pseudomonas sp. ST41	0	0	0	0	1	0	0
Pseudomonas sp. SY4	1	0	0	0	0	0	0
Pseudomonas mosselii	0	0	0	0	0	0	0
Pseudomonas reactans	0	0	0	0	0	1	0
Pseudomonas sp. AC2	1	0	0	0	0	0	0
Pseudomonas sp. AR(2010)	1	0	0	0	0	0	0
Pseudomonas sp. C6-9	1	0	0	0	0	0	0
Pseudomonas sp. CMR5c	1	0	0	0	0	0	0
Pseudomonas sp. S9	0	0	0	0	0	0	1

Pseudomonas thermotolerans	1	0	0	0	0	0	0
Pseudomonas oryzihabitans	0	0	0	0	0	0	0
Pseudomonas pertucinogena	1	0	0	0	0	0	0
Pseudomonas sp. PT03	0	0	0	0	0	1	0
Pseudomonas sp. R125	1	0	0	0	0	0	0
Pseudomonas sp. SK38	1	0	0	0	0	0	0
Pseudomonas sp. TAC II 18	0	0	0	0	0	0	0
Pseudomonas wisconsinensis	0	0	0	0	0	0	1
Pseudomonas azotifigens	0	0	0	0	1	0	0
Pseudomonas mucidolens	0	0	0	0	0	0	0
Pseudomonas sp. ED3	0	0	0	1	0	0	0

Prokaryotes abundance, ***Azotobacter*** species (4 OTUs)

	Swine wastewater	SS-inoculated MFC, Anode	SS-inoculated MFC, Planktonic	IGBS-inoculated MFC, Anode	IGBS-inoculated MFC, Planktonic	IGBS	Swine sludge
Azotobacter vinelandii	2448	1567	450	3387	3517	641	1638
Azotobacter chroococcum	20	32	10	99	52	6	24
Azotobacter sp. FA8	0	3	0	3	11	0	5
Azotobacter salinestris	0	1	0	4	4	0	3

Prokaryotes abundance, ***Xanthomonas*** species (20 OTUs)

	Swine wastewater	SS-inoculated MFC, Anode	SS-inoculated MFC, Planktonic	IGBS-inoculated MFC, Anode	IGBS-inoculated MFC, Planktonic	IGBS	Swine sludge
Xanthomonas campestris	1248	2204	527	2849	2243	379	642
Xanthomonas oryzae	671	1303	338	1693	1406	276	357
Xanthomonas citri	667	1161	267	1542	1293	195	341
Xanthomonas albilineans	427	875	194	1355	1041	128	227
Xanthomonas euvesicatoria	548	806	209	1109	950	147	258
Xanthomonas fuscans	221	473	93	622	505	70	134
Xanthomonas axonopodis	9	35	3	21	21	1	4
Xanthomonas vesicatoria	6	8	0	8	7	0	3
Xanthomonas sp. T22	0	1	0	1	2	3	1
Xanthomonas perforans	2	3	0	1	1	0	1
Xanthomonas vasicola	1	1	0	0	2	0	0
Xanthomonas translucens	0	0	0	0	1	0	0
Xanthomonas sp. CIP 102397	0	0	0	1	0	0	0
Xanthomonas gardneri	0	1	0	0	0	0	0

Prokaryotes abundance, ***Shewanella*** species (25 OTUs)

	Swine wastewater	SS-inoculated MFC, Anode	SS-inoculated MFC, Planktonic	IGBS-inoculated MFC, Anode	IGBS-inoculated MFC, Planktonic	IGBS	Swine sludge
Shewanella baltica	1770	585	331	747	588	1676	632
Shewanella sp. W3-18-1	1688	182	97	374	317	2337	583
Shewanella oneidensis	676	234	164	324	332	1133	304
Shewanella putrefaciens	556	225	100	357	208	1429	210
Shewanella sediminis	616	425	296	425	532	416	312
Shewanella amazonensis	727	333	248	390	479	366	299
Shewanella pealeana	520	326	181	338	448	325	279
Shewanella frigidimarina	634	298	171	351	346	275	258
Shewanella sp. ANA-3	619	260	119	287	237	478	232
Shewanella loihica	506	279	191	321	357	265	255
Shewanella denitrificans	596	228	144	310	324	193	238
Shewanella woodyi	333	172	150	196	265	199	152
Shewanella sp. MR-7	315	118	85	160	162	361	140
Shewanella halifaxensis	244	179	96	172	199	171	133
Shewanella piezotolerans	261	126	94	139	198	175	110
Shewanella sp. MR-4	200	125	83	129	125	270	108
Shewanella violacea	272	84	64	88	106	190	135
Shewanella benthica	218	91	49	91	116	120	97
Shewanella sp. 33B	0	4	0	9	35	1	0
Shewanella livingstonensis	7	0	0	0	0	0	2
Shewanella hanedai	0	3	2	0	1	1	1
Shewanella algae	1	0	0	0	0	0	1
Shewanella sp.	0	0	0	0	0	1	0
Shewanella colwelliana	0	0	0	0	0	1	0
Shewanella sp. DB6705	0	0	0	0	0	0	0

Prokaryotes abundance, ***Methylobacter*** species (3 OTUs)

	Swine wastewater	SS-inoculated MFC, Anode	SS-inoculated MFC, Planktonic	IGBS-inoculated MFC, Anode	IGBS-inoculated MFC, Planktonic	IGBS	Swine sludge
Methylobacter tundripaludum	180	4363	511	5889	8092	495	184
Methylobacter marinus	0	0	0	2	3	1	0
Methylobacter bovis	0	0	0	0	0	0	0

Total hit numbers of class ***Epsilonproteobacteria***

	Swine wastewater	SS-inoculated MFC, Anode	SS-inoculated MFC, Planktonic	IGBS-inoculated MFC, Anode	IGBS-inoculated MFC, Planktonic	IGBS	Swine sludge
Epsilonproteobacteria	45137	24913.25	15848	26223	61311.25	4260	20853

Prokaryotes abundance, ***Arcobacter*** species (11 OTUs)

	inflowSW	MFC1_a	MFC1_p	MFC3_a	MFC3_p	rIS	rSW
Arcobacter butzleri	27488	4304	3454	5211	25344	185	12601
Arcobacter nitrofigilis	4289	887	737	1026	3301	107	1861
Arcobacter cryaerophilus	30	4	9	0	13	0	12
Arcobacter sp. L	13	2	2	2	11	0	8
Arcobacter skirrowii	20	1	1	1	1	0	7
Arcobacter cibarius	9	0	2	0	1	0	2
Arcobacter cf. butzleri LCDC13432	2	0	0	0	1	0	0
Arcobacter trophiarum	3	0	0	0	0	0	1
Arcobacter defluvii	0	0	0	0	2	0	0
Arcobacter cf. butzleri LCDC13207	2	0	0	0	0	0	0
Arcobacter mytili	0	0	0	0	1	0	0

Total hit numbers of phylum ***Firmicutes***

	Swine wastewater	SS-inoculated MFC, Anode	SS-inoculated MFC, Planktonic	IGBS-inoculated MFC, Anode	IGBS-inoculated MFC, Planktonic	IGBS	Swine sludge
Firmicutes	841540.5	229422.25	219750.5	143203.5	151085.25	119895.5	574808

Prokaryotes abundance, ***Clostridium*** species (77 OTUs)

	Swine wastewater	SS-inoculated MFC, Anode	SS-inoculated MFC, Planktonic	IGBS-inoculated MFC, Anode	IGBS-inoculated MFC, Planktonic	IGBS	Swine sludge
Clostridium botulinum	15215	5154	5083	3010	4017	2094	10268
Clostridium thermocellum	9754	6740	5567	3559	3574	2253	7088
Clostridium phytofermentans	14049	3200	2844	1691	1491	621	7910
Clostridium beijerinckii	11976	2782	3020	1529	2429	895	7981
Clostridium saccharolyticum	12916	2889	2459	1477	1203	386	8155
Clostridium perfringens	7065	2282	2271	1275	1924	916	4647
Clostridium kluyveri	6266	2782	2389	1563	1575	910	4642
Clostridium leptum	6471	1840	3113	946	1032	328	5926
Clostridium cellulolyticum	4923	3003	2456	1568	1588	771	3435
Clostridium acetobutylicum	5098	1665	1629	1088	1179	670	3406
Clostridium bolteae	4902	1131	983	693	615	297	4179
Clostridium cellulovorans	4309	1552	1480	833	928	446	2932
Clostridium asparagiforme	4047	1369	1074	794	673	434	2571
Clostridium tetani	3180	1488	1361	885	955	704	2192

Clostridium novyi	2979	1392	1281	775	935	618	2043
Clostridium hathewayi	3665	880	729	492	420	213	2466
Clostridium ljungdahlii	2821	1121	955	683	762	506	2001
Clostridium butyricum	3138	802	958	388	880	144	2455
Clostridium nexile	4169	572	595	278	289	112	2389
Clostridium methylpentosum	2690	1206	1151	635	564	215	1867
Clostridium scindens	3484	707	637	388	346	177	2423
Clostridium sp. M62/1	3513	714	667	405	337	127	2058
Clostridium sp. 7_2_43FAA	2441	834	947	363	1003	195	1752
Clostridium carboxidivorans	1892	1117	930	699	773	579	1466
Clostridium papyrosolvens	1602	1275	944	685	686	347	1056
Clostridium symbiosum	2662	757	624	364	354	142	1690
Clostridium hylemonae	2390	700	547	392	329	199	1628
Clostridium sp. L2-50	2347	428	418	243	212	78	1230
Clostridium sp. SS2/1	2146	367	394	199	213	90	1191
Clostridium sporogenes	366	217	216	129	181	123	284
Clostridium sp. HGF2	556	135	141	86	67	29	375
Clostridium citroniae	141	35	47	19	13	0	80
Clostridium pasteurianum	84	29	23	20	21	17	64
Clostridium sp. enrichment culture clone 7-14	116	37	19	18	17	2	47
Clostridium saccharobutylicum	49	18	15	13	18	13	27
Clostridium subterminale	18	32	22	24	32	15	9
Clostridium sp. D5	65	6	11	3	5	0	36
Clostridium stercorarium	41	11	10	11	7	10	15
Clostridium tetanomorphum	19	16	15	7	6	4	12
Clostridium aminobutyricum	4	13	10	10	10	14	11
Clostridium sp. 7_3_54FAA	30	3	4	2	5	0	11
Clostridium clariflavum	6	13	9	3	4	2	9
Clostridium clostridioforme	23	1	4	1	1	0	15
Clostridium longisporum	18	1	2	0	3	0	15
Clostridium sp. enrichment culture clone 7-25	16	2	2	2	2	0	11
Clostridium sartagoforme	17	0	1	1	0	5	5
Clostridium scatologenes	3	6	5	3	1	4	3
Clostridium saccharoperbutylace-tonicum	8	3	2	4	3	0	2
Clostridium tyrobutyricum	4	3	2	8	2	1	3
Clostridium septicum	7	1	2	0	1	0	6
Clostridium cochlearium	5	3	4	0	0	1	1
Clostridium histolyticum	3	1	1	0	3	3	1
Clostridium magnum	1	2	1	1	2	0	5
Clostridium cylindrosporum	4	0	2	0	1	0	5
Clostridium sp. M-E	1	3	1	2	1	1	1
Clostridium propionicum	2	1	1	0	1	1	3
Clostridium hungatei	0	3	0	4	1	0	0
Clostridium sp.	3	0	1	0	0	1	1
Clostridium josui	1	0	0	1	1	1	3
Clostridium cellobioparum	2	2	0	1	0	0	1
Clostridium argentinense	0	0	0	0	1	2	0
Clostridium aminovalericum	2	0	1	0	0	0	1
Clostridium acidurici	0	0	0	0	0	1	1
Clostridium limosum	0	1	0	0	1	0	0
Clostridium sp. MCF-1	1	0	0	0	0	0	1
Clostridium paraputrificum	0	0	0	0	0	0	1
Clostridium sp. Kas107-2	1	0	0	0	0	0	0
Clostridium cadaveris	0	0	0	0	0	0	0
Clostridium diolis	0	0	0	0	0	1	0
Clostridium chauvoei	1	0	0	0	0	0	0

Clostridium ultunense	0	0	0	0	0	0	0
Clostridium aceticum	0	0	0	0	0	0	0
Clostridium sp. CA6	0	0	0	0	0	0	0
Clostridium sp. DL-VIII	1	0	0	0	0	0	0

Total hit numbers of phylum ***Bacteroidetes***

	Swine wastewater	SS-inoculated MFC, Anode	SS-inoculated MFC, Planktonic	IGBS-inoculated MFC, Anode	IGBS-inoculated MFC, Planktonic	IGBS	Swine sludge
Bacteroidetes	953059	177280.25	259630.25	125597.5	148800.25	44250.5	300106

Prokaryotes abundance, ***Flavobacterium*** species (15 OTUs)

	Swine wastewater	SS-inoculated MFC, Anode	SS-inoculated MFC, Planktonic	IGBS-inoculated MFC, Anode	IGBS-inoculated MFC, Planktonic	IGBS	Swine sludge
Flavobacterium johnsoniae	7892	4405	5334	3969	4264	971	2843
Flavobacterium psychrophilum	3370	2710	3497	2333	2537	489	1412
Flavobacterium columnare	11	9	16	7	9	1	9
Flavobacterium sp.	9	8	5	5	5	2	6
Flavobacterium sp. 4214	4	1	5	0	2	1	3
Flavobacterium sp. 4H	3	1	5	1	5	0	0
Flavobacterium hibernum	1	2	1	0	2	0	2
Flavobacterium frigidimaris	3	0	0	1	1	0	0
Flavobacterium sp. KP1	4	0	0	0	0	0	0
Flavobacterium aquatile	1	0	0	0	0	0	0
Flavobacterium sp. TN17	1	0	0	0	0	0	0
Flavobacterium frigoris	0	0	0	0	0	0	0
Flavobacterium sp. 4221	0	0	0	0	0	1	0
Flavobacterium sp. 92	0	1	0	0	0	0	0
Flavobacterium sp. LW53	0	0	0	0	0	0	1

Prokaryotes abundance, ***Bacteroides*** species (45 OTUs)

	Swine wastewater	SS-inoculated MFC, Anode	SS-inoculated MFC, Planktonic	IGBS-inoculated MFC, Anode	IGBS-inoculated MFC, Planktonic	IGBS	Swine sludge
Bacteroides fragilis	40447	7677	10712	5096	5533	1517	13746
Bacteroides vulgatus	33183	3715	6287	2466	2832	760	9948
Bacteroides sp. 1_1_6	19812	3147	4713	2134	2398	607	6318
Bacteroides sp. 4_3_47FAA	19687	2910	4782	1927	2194	446	5938
Bacteroides thetaiotaomicron	17194	2848	4051	1906	2032	567	5585
Bacteroides helcogenes	16184	2227	3480	1542	1613	361	5264
Bacteroides sp. 20_3	9379	3310	4348	2131	2384	694	3827
Bacteroides cellulosilyticus	9959	1583	2241	1085	1170	412	3178
Bacteroides intestinalis	8839	1462	2082	985	1064	295	2764
Bacteroides coprocola	8966	1397	2239	888	1010	228	2750
Bacteroides plebeius	8516	1420	2327	912	1029	266	2827
Bacteroides eggerthii	6611	1148	1639	750	825	246	2224
Bacteroides ovatus	7418	819	1268	651	612	152	2289
Bacteroides uniformis	6176	1104	1539	761	824	224	1992
Bacteroides sp. D2	6673	771	1111	622	623	152	1978
Bacteroides coprophilus	5515	1066	1542	702	782	219	1883
Bacteroides sp. 2_2_4	6293	817	1191	594	567	128	1984
Bacteroides stercoris	4777	942	1337	627	664	139	1661
Bacteroides finegoldii	5075	666	974	500	516	112	1670
Bacteroides sp. D20	4213	773	1034	546	556	172	1391
Bacteroides caccae	4289	727	959	519	531	165	1408
Bacteroides sp. D1	4395	576	860	399	437	75	1346
Bacteroides sp. 2_1_56FAA	3658	717	984	450	558	164	1335
Bacteroides dorei	4364	526	838	349	348	89	1281
Bacteroides sp. 9_1_42FAA	3907	515	899	406	416	97	1285
Bacteroides sp. 1_1_14	3248	523	796	363	430	128	1063
Bacteroides sp. 2_1_16	2728	586	819	373	415	105	935
Bacteroides sp. 2_1_33B	2181	652	833	413	484	130	893
Bacteroides sp. 3_1_33FAA	1961	179	345	131	141	49	576
Bacteroides sp. 2_1_7	1460	281	414	189	208	75	499
Bacteroides sp. D22	1700	162	251	120	113	30	533
Bacteroides sp. 3_1_23	1544	165	212	129	116	37	484
Bacteroides sp. 3_1_19	1098	279	341	158	195	75	444
Bacteroides sp. 3_2_5	1076	272	362	157	164	53	353
Bacteroides xylanisolvens	950	143	205	113	100	30	302
Bacteroides sp. 4_1_36	859	105	138	66	71	17	291
Bacteroides sp. 3_1_40A	786	50	88	33	37	8	216
Bacteroides sp. 1_1_30	443	66	113	45	46	2	144
Bacteroides coprosuis	263	46	55	14	20	0	74
Bacteroides sp. 2_1_22	216	18	36	18	14	5	60
Bacteroides fluxus	34	5	4	2	3	0	4
Bacteroides sp.	1	0	1	0	1	2	0
Bacteroides sp. WA1	1	0	0	0	0	0	1
Bacteroides graminisolvens	0	0	0	0	0	0	0
Bacteroides propionicifaciens	0	0	0	0	0	0	0

Prokaryotes abundance, ***Parabacteroides*** species (4 OTUs)

	Swine wastewater	SS-inoculated MFC, Anode	SS-inoculated MFC, Planktonic	IGBS-inoculated MFC, Anode	IGBS-inoculated MFC, Planktonic	IGBS	Swine sludge
Parabacteroides distasonis	23579	7320	9467	4863	5299	1546	9982
Parabacteroides johnsonii	8714	2707	3383	1827	1975	552	4139
Parabacteroides merdae	7966	2523	3117	1764	1860	537	3876
Parabacteroides sp. D13	8131	2594	3269	1798	1873	552	3300

Total hit numbers of domain ***Archaea***

	Swine wastewater	SS-inoculated MFC, Anode	SS-inoculated MFC, Planktonic	IGBS-inoculated MFC, Anode	IGBS-inoculated MFC, Planktonic	IGBS	Swine sludge
Archaea	14263	84409.25	61551.75	85687.5	125856	85814.5	42164.5

Prokaryotes abundance, ***Methanosarcina*** species (4 OTUs)

	Swine wastewater	SS-inoculated MFC, Anode	SS-inoculated MFC, Planktonic	IGBS-inoculated MFC, Anode	IGBS-inoculated MFC, Planktonic	IGBS	Swine sludge
Methanosarcina barkeri	876	20354	10729	2693	6296	2378	11534
Methanosarcina acetivorans	673	4801	2980	2533	4230	2897	2755
Methanosarcina mazei	541	4683	2765	1720	3176	2302	3133
Methanosarcina thermophila	7	117	70	45	92	62	76

Prokaryotes abundance, ***Methanothermobacter*** species (3 OTUs)

	Swine wastewater	SS-inoculated MFC, Anode	SS-inoculated MFC, Planktonic	IGBS-inoculated MFC, Anode	IGBS-inoculated MFC, Planktonic	IGBS	Swine sludge
Methanothermobacter thermautotrophicus	385	1878	1494	14287	19238	7214	1010
Methanothermobacter marburgensis	114	876	689	8024	10747	3944	390
Methanothermobacter wolfeii	0	4	2	17	23	6	1

Prokaryotes abundance, ***Methanosaeta*** species (3 OTUs)

	Swine wastewater	SS-inoculated MFC, Anode	SS-inoculated MFC, Planktonic	IGBS-inoculated MFC, Anode	IGBS-inoculated MFC, Planktonic	IGBS	Swine sludge
Methanosaeta thermophila	246	3646	2346	5375	12650	8927	2688
Methanosaeta harundinacea	7	115	87	244	522	456	90
Methanosaeta concilii	4	42	25	62	131	95	33

Prokaryotes abundance, ***Methanospirillum*** species (2 OTUs)

	Swine wastewater	SS-inoculated MFC, Anode	SS-inoculated MFC, Planktonic	IGBS-inoculated MFC, Anode	IGBS-inoculated MFC, Planktonic	IGBS	Swine sludge
Methanospirillum hungatei	591	10919	7019	3368	3598	4102	2415
Methanospirillum lacunae	0	1	0	0	0	0	0
